# Aspects of Wellbeing for Indigenous Youth in CANZUS Countries: A Systematic Review

**DOI:** 10.3390/ijerph192013688

**Published:** 2022-10-21

**Authors:** Kate Anderson, Elaina Elder-Robinson, Alana Gall, Khwanruethai Ngampromwongse, Michele Connolly, Angeline Letendre, Esther Willing, Zaine Akuhata-Huntington, Kirsten Howard, Michelle Dickson, Gail Garvey

**Affiliations:** 1 School of Public Health, The University of Queensland, Herston, QLD 4006, Australia; 2 Menzies School of Health Research, Charles Darwin University, Casuarina, NT 0810, Australia; 3 International Group for Indigenous Health Statistics, Columbia, MD 21045, USA; 4 Alberta Cancer Prevention Legacy Fund, Population, Public and Indigenous Health, Alberta Health Services, 102 Anderson Hall, 10959 102 ST NW, Edmonton, AB T5H 3V9, Canada; 5 Kōhatu–Centre for Hauora Māori, University of Otago, Dunedin 9054, New Zealand; 6 School of Public Health, Faculty of Medicine and Health, The University of Sydney, Sydney, NSW 2006, Australia; 7 Menzies Centre for Health Policy and Economics, Faculty of Medicine and Health, University of Sydney, Sydney, NSW 2006, Australia

**Keywords:** Indigenous health and wellbeing, First Nations, Indigenous peoples, wellbeing, culture, quality of life, QoL

## Abstract

Indigenous children and young people (hereafter youth) across CANZUS nations embody a rich diversity of cultures and traditions. Despite the immense challenges facing these youth, many harness cultural and personal strengths to protect and promote their wellbeing. To support this for all youth, it is critical to understand what contributes to their wellbeing. This review aims to identify components contributing to wellbeing for Indigenous youth in CANZUS nations. Five databases were searched from inception to August 2022. Papers were eligible if they: focused on Indigenous youth in CANZUS nations; included views of youth or proxies; and focused on at least one aspect of wellbeing. We identified 105 articles for inclusion (Canada *n* = 42, Australia *n* = 27, Aotearoa New Zealand *n* = 8, USA *n* = 28) and our analysis revealed a range of thematic areas within each nation that impact wellbeing for Indigenous youth. Findings highlight the unique challenges facing Indigenous youth, as well as their immense capacity to harness cultural and personal strengths to navigate into an uncertain future. The commonalities of Indigenous youth wellbeing across these nations provide valuable insights into how information and approaches can be shared across borders to the benefit of all Indigenous youth and future generations.

## 1. Introduction

There are more than 370 million Indigenous peoples around the world with diverse cultures and ways of life [1]. Canada, Australia, Aotearoa New Zealand and the United States of America (USA), collectively known as CANZUS nations [2], share an enduring legacy of European colonisation. There are many diverse Indigenous nations across these countries, each distinct in their rich cultural identities and knowledge systems, known as: First Nations and First Peoples (terms used across CANZUS nations); Aboriginal and Torres Strait Islander (Australia); Māori (Aotearoa New Zealand); First Nations, Métis and Inuit (Canada); and American Indian, Alaska Native and Native Hawaiian (USA) [2]. While acknowledging the great diversity among the Indigenous peoples in CANZUS nations, in this paper we respectfully use the term ‘Indigenous peoples’ to reference all Indigenous peoples across CANZUS nations, and ‘Indigenous groups’ when referring to multiple Indigenous population groups across a nation. While there are clear unique and diverse beliefs, cultural practices, and geographic settings between Indigenous peoples and groups across CANZUS nations, there are some similarities in Indigenous worldviews. Informed by connections to community, and the land and seas of their environments, Indigenous groups commonly hold holistic and collectivist conceptions of health and wellbeing [3]. The shared histories of displacement, discrimination and disadvantage as a result of colonial activities on the land and seas of CANZUS nations [4,5]. and the implementation of institutional systems, has disrupted Indigenous peoples ways of knowing, being and doing [2]. Such forces are known to negatively impact Indigenous peoples’ health and wellbeing [3].

Indigenous children and adolescents (hereafter referred to as *youth*) account for a greater proportion of the Indigenous populations in CANZUS countries, compared to their non-Indigenous populations (Indigenous versus non-Indigenous median age: Canada, 32.1 years versus 40.9 years [6]; Australia, 20.3 years versus 37.8 years [7]; Aotearoa New Zealand, 26.1 years versus 37.5 years [8]; United States, 32.9 versus 38.5 years) [9]. While youth in CANZUS nations are generally among the healthiest in the world, significant health inequities exist for Indigenous youth in these nations [10,11]. In addition to the unprecedented environmental, social and technological changes facing all youth, Indigenous youth face additional challenges associated with ongoing intergenerational trauma, racism and socioeconomic disadvantage [12,13,14]. The substantial burden of these and other challenges is reflected in the higher rates of psychological distress, depression, anxiety, substance abuse, self-harm and suicide among Indigenous youth compared to non-Indigenous youth in CANZUS nations [10,11,12,13,15,16]. Further, displacement of Indigenous youth from family, community, and Country, Lands or Nation is seen in higher rates of incarceration or detention [17,18,19], placement in out-of-home care or child welfare systems [20,21,22,23], and homelessness [24], compared to their non-Indigenous counterparts in CANZUS nations.

To effectively support Indigenous youth to overcome the challenges they face and to harness their own cultural and personal strengths to thrive, it is critical to understand and be able to assess their wellbeing status, using metrics and measures that ask about the parts of life that are important and relevant to them. Readily available measures of wellbeing allow for early identification of issues for youth and to develop and evaluate effective interventions and service delivery [25]. To develop such measures, it is first necessary to identify the parts of life that are important and that influence and shape wellbeing for Indigenous youth. While wellbeing is variably defined, the Centers for Disease Control and Prevention describes wellbeing as a subjective measure that “*can be described as judging life positively and feeling good*” [26]. What makes up these subjective experiences of wellbeing is culturally bound [27,28]. Therefore, wellbeing from an Indigenous worldview varies significantly from Western biomedically informed perspectives [27]. 

Understanding and measuring wellbeing of Aboriginal and Torres Strait Islander Australian adults has been a research focus for our research team over the past seven years [27,28,29,30,31]. Literature reviews conducted by our team have explored wellbeing for Indigenous adults across CANZUS countries, identifying aspects of wellbeing that were unique to each country, as well as some commonalities [27,28]. Additionally, our team has conducted qualitative research that has elicited rich data about the foundations of Aboriginal and Torres Strait Islander Australian adults’ wellbeing [29], which was used to inform the development of nationally relevant What Matters 2 Adults wellbeing measure (WM2A) [31]. While the attention on Indigenous adults’ wellbeing is important, it does not necessarily reflect the nature and aspects of the wellbeing of Indigenous youth.

There is a pressing need to identify and explore what parts of life are of importance to the wellbeing of Indigenous youth. The aim of this systematic literature review is to identify and describe the existing evidence base around the parts of life that are important to the wellbeing of Indigenous youth in CANZUS nations.

## 2. Materials and Methods

This review was led by a senior non-Indigenous researcher (KA), with assistance from a non-Indigenous researcher (EE) and two Indigenous Australian researchers (AG, KN). The review was overseen by senior Indigenous researchers from Canada (AL), Australia (GG, MD), Aotearoa New Zealand (EW, ZA) and the USA (MC). This review forms part of a larger body of work, the What Matters Research Program, that aims to develop new, nationally and culturally relevant measures to assess wellbeing in Aboriginal and Torres Strait Islander peoples (What Matters 2 Adults—18 years and older; What Matters 2 Youth—12–17 years; What Matters 2 Kids—5–11 years), with the adult measure completed and currently in pilot implementation stage [29,31]. Indigenous Project Advisory Groups in Australia have been formed to guide the What Matters 2 Adults [29,31] What Matters 2 Youth and What Matters 2 Kids projects. 

### 2.1. Protocol Registration

A protocol for this systematic review was published on PROSPERO: International prospective register of systematic reviews website [Registration number: CRD42020206944].

### 2.2. Eligibility Criteria

Eligible articles included qualitative and mixed methods studies that reported on at least one identifiable, substantial aspect of wellbeing in youth (aged 18 years or younger) identifying as Indigenous in one of the four CANZUS nations. 

Studies where the age range of participants was above the age cut-off were included if a majority of participants were 18 years old or younger. No lower age limit was applied to the review with the intention to explore youth wellbeing at all ages. As some youth (e.g., those of younger age or those with learning delays/difficulties) may be unable to convey the full experience of their wellbeing, studies using a ‘proxy’ to report on youth wellbeing were also included in this review (proxy includes any significant Indigenous or non-Indigenous adult figures in the youths’ lives including parents, caregivers, teachers and healthcare workers). Studies reporting on youth perspectives were included only if youth were identified as Indigenous; perspectives from proxy participants were included if they reported on Indigenous youth wellbeing, regardless of the proxy’s Indigenous status. 

We excluded articles where findings were reported in relation to a specific disease or condition and where the focus of the study was the wellbeing of adults. Medical case studies, case series, commentary, editorial, opinion papers, books and book chapters, conference abstracts, dissertations, theses and other grey literature were excluded. 

### 2.3. Search Strategy

We searched titles and abstracts in the databases APA PsycInfo, CINAHL, Medline, Embase and PubMed, with no date limits. The initial search was run in July 2020, and updated in January 2022. Search terms were developed through the inclusion of terms used in previous systematic reviews with an Indigenous [27] and youth focus [32,33,34], in addition to consultation with country-specific experts (GG, EW, MC, AL). Key search terms were: (a) Indigenous peoples from CANZUS countries; (b) quality of life and wellbeing terms and; (c) youth terms. Limiters used included studies with humans and peer-reviewed, where available in each databases interface. A Google Scholar search was used, with the first ten pages of search results scanned, to identify any further eligible papers for inclusion. Additional Indigenous specific databases were reviewed for eligible articles, upon recommendation from country specific experts, including: Circumpolar Health Bibliographic Database; Native Health Database; Arctic Health Publications Database; International Journal of Indigenous Health; and Journal of Aboriginal Health. An example search strategy is presented in Table 1 and the complete strategy is provided in Supplement S1.

### 2.4. Study Selection

**Figure 1:**
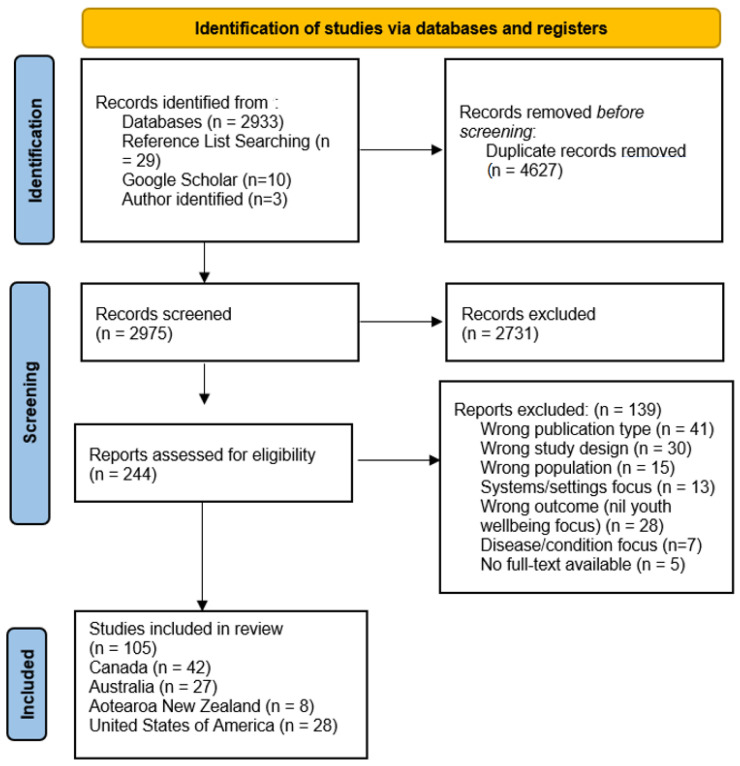
Preferred Reporting Items for Systematic Reviews and Meta-Analyses (PRISMA) Flowchart.

Duplicate articles were removed using EndNote software [35]. Two reviewers (EE, KA) then undertook title and abstract screening of studies in Rayyan Online Software [36] using a specified screening hierarchy (Supplement S2) to assist with inclusion and exclusion decisions. After deduplication, approximately ten percent of the total articles (number of articles = 2975) were title/abstract screened independently by both reviewers, with any conflicts resolved through discussion to improve consistency of screening. Each reviewer then screened half of the remaining articles, by title/abstract, independently. This process was repeated at full-text review (number of articles = 244). The reference lists of included articles, and relevant reviews identified in the initial search (number of articles = 21, Supplement S3), were assessed for further relevant articles. Figure 1 shows reasons for exclusion and the final number of included articles (*n* = 105).

### 2.5. Data Collection and Analysis

Data extraction was undertaken by two reviewers (EE, KN) and cross-checked by a senior reviewer (KA). Headings used to extract data, where available, included: publication information (authors, year published, study location, study methods, aim of study) and participant details (specific Indigenous group, setting, number of participants, participant age, number of Indigenous participants, gender distribution).

Included studies were uploaded into NVivo12 qualitative analysis software [37] and grouped by country. Thematic analysis was used throughout the review, to analyse each country separately [38]. The results section of each article was reviewed line-by-line by reviewers (KA, EE, KN), with results coded to country-specific themes. Reviewers coded approximately twenty percent of the studies in each country group independently, meeting to ensure consistency of extracted themes and to confirm codes. Remaining articles were then split between reviewers (EE, KA, KN), who met regularly to discuss and consolidate the developing themes. Findings were drafted into country specific results (EE, KA, KN, AG), with major themes functioning as headings for each results section. These draft country-specific results were shared with Indigenous co-authors in each country (GG, MD, AL, MC, EW, ZA), who provided feedback and shared expertise, and results were revised accordingly.

## 3. Results

### 3.1. Paper Characteristics

Of 105 included articles, from 98 unique studies, 42 (40%) were from Canada, 27 (26%) from Australia, 8 (8%) from Aotearoa New Zealand and 28 (27%) from the USA (see Table 2). Ninety-six studies (91%) used only qualitative methods, with nine (9%) using mixed-methods, and most using community and researcher networks to recruit participants. A third of studies (*n* = 34; 32%) had a wellbeing focus, whilst 71 (68%) studies discussed wellbeing themes alongside other themes.

Most studies (*n* = 78; 74%) were conducted with Indigenous participants only, with 21 (20%) studies having a mix of Indigenous and non-Indigenous participants, and six (6%) studies not reporting participant characteristics. Indigenous participants’ views have been prioritised throughout our analysis.

Participant ages were mixed: 37 studies (35%) included youth only participants; 32 studies (30%) included a combination of youth and adult proxy participants; and 36 studies (36%) included adult proxy participants only. The views of both youth and proxy participants have been included in the current review.

### 3.2. Thematic Synthesis

The CANZUS nations represent four individual, present-day nation states, however these borders are not always reflective of Indigenous nations, communities and tribes that reside within, and in the case of Canada and the USA, across them. In the current review, results have been thematically grouped by each CANZUS nation. To capture the complexities of such circumstances, this review presents results within each nation state and between them. Exemplary quotes reflecting the content in each of the themes are presented in Appendix A—Nation-Specific Themes and Exemplar Quotes.

#### 3.2.1. Indigenous Youth in Canada

Indigenous peoples in Canada comprise three culturally distinct groups: First Nations, Inuit and Métis. There are more than 630 First Nations communities in Canada. Inuit peoples reside in the artic regions of northern Canada. Métis are a distinct Indigenous people who emerged after European influence on Canadian lands. Indigenous peoples in Canada reside in a number of locations, including Reserves and First Nations communities, as well as urban and regional centres. Negotiations around land claims, Indigenous rights, and treaties continue between Indigenous peoples in Canada and the Government of Canada [39].

Forty-two articles [40,41,42,43,44,45,46,47,48,49,50,51,52,53,54,55,56,57,58,59,60,61,62,63,64,65,66,67,68,69,70,71,72,73,74,75,76,77,78,79,80,81], from 38 unique studies, reported on aspects of Indigenous youth wellbeing in Canada. Our thematic analysis identified eight aspects of wellbeing for this population: basic resources for survival; safety and stability; relationships with others; culture and spirituality; knowledge, opportunities, and the future; identity; resilience and independence; and recreation and interests. These studies reveal that the wellbeing of Indigenous youth in Canada is dependent on achieving a complex and precarious balance. This young population is described as striving to embody and maintain their traditional culture, within the context of intergenerational and cumulative trauma, while navigating into an uncertain future.

**Table 2:** Included articles, details and participants characteristics.

Authors (Year)	Region	Study Setting	Indigenous Group	Participant Details	Reporting Person (Youth, Family Proxy, Service Provider Proxy)	Brief Methods	Was Wellbeing Part of Main Aim (YES) or Component of the Broader Research Question (BROAD)?
**CANADA**							
Ansloos et al. (2021) [67]	Vancouver	Community	Indigenous	8 participants (5 Indigenous participants—analysis only of Indigenous participants) 3F 1M 1Two-Spirit 16–25 years	Youth, retrospective youth	Interviews and observations	BROAD
Aylward et al. (2015) [40]	Nunavut	Regional youth program	Nunavut Inuit	10 Indigenous participants. 5F 5M Alumni who had completed the Northern Youth Abroad Program 2006–2011	Youth	Semi-structured interviews	BROAD
Berman et al. (2009) [41]	South Ontario	Community	NR	6 Aboriginal participants, out of 19—Aboriginal participant contributions specified All F 14–19 years	Youth	Adapted ethnographic study (field notes and interview style discussion)	BROAD
Brown et al. (2012) [42]	Alert Bay	Community	Namgis First nation	Participant details not reported	Youth and Elders	Individual interviews, focus groups	YES
Clark et al. (2013) [82]	Kamloops, British Columbia	Community	Melq’ilwiye	40 Indigenous participants 24F 16M 12–15 years	Youth	Talking circles (40 participants) and surveys	YES
Gerlach et al. (2018) [44]	British Columbia	Community services	NR	35 participants (10 caregivers, 18 workers, 4 Elders, 3 administrative leaders) 30F 2M (excluding administrative leaders)	Indigenous caregivers (mothers, aunties, fathers, Elders) & Aboriginal Infant Development Program workers	In-depth individual and small group interviews	BROAD
Hardy et al. (2020) [68]	Toronto	Community	NR	12 Indigenous participants All self-identified 2SLGBTTQQIA youth	Youth	Focus groups (7 participants) and surveys (5 participants)	BROAD
Hatala et al. (2017) [45] Hatala et al. (2019) [77] Hatala et al. (2020) [78] Njeze et al. (2020) [70]	Saskatoon	Community	Plains Cree, Métis Nêhiyaw (Plains Cree), Métis Plains Cree, Métis Nêhiyaw (Cree), Métis, Dene	28 Indigenous participants 15–25 years 28 Indigenous participants 16F 12M 15–25 years 28 Indigenous participants 16F 12M 16–25 years 6 Indigenous youth (selected from above cohort) 3F 3M	Youth	Photovoice and photo elicitation with open talking circle discussions/ interviews. Four rounds over the course of a year	YES
Isaak et al. (2008) [46]	Northern Manitoba	Community	Northern Manitoba First nations	39 participants (10 adults, 29 children) Children: 13F 16M Children: 12–19 years; Adults: 21–89 years	Youth and proxy reporters (teachers, youth counsellors, community members, Elders, health workers and health board members)	Individual in-depth interviews w/adults; focus groups w/youth	YES
Kral (2013) [47]	Igloolik	Community	Inuit	27 Indigenous participants 11F 15M 17–24 years: 9; 25–44 years: 9; 45+ years: 9	Youth and proxy community members	Open-ended interviews	YES
Kral et al. (2011) [48]	Nunavut	Community	Igloolik, Qikiqtarjuaq	50 Indigenous participants 25F 25M 14–94 years	Youth and Elders (responses not separated)	Open-ended interviews and surveys	YES
Kyoung et al. (2015) [49]	Edmonton	Community	NR	53 participants (8 Indigenous) 36F 17M 18–51 years	Key informants (44 responsible for care of Aboriginal youths)	Semi-structured interviews, field notes and memos	YES
Latimer et al. (2020) [50]	Atlantic region	Community & service delivery	Mi’kmaq, Wolastoq	220 participants (189 Indigenous community members, 32 professionals in the community; 146 youth participants). Youth: grades 1–12	Youth, parents and Elders, adult professionals in the community	Semi-structured conversation sessions and interview sessions	BROAD
Liebenberg et al. (2022) [79]	Atlantic Canada	Community & service delivery	First Nations	8 Indigenous participants 14–18 years	Youth	Photovoice, videography, focus group.	YES
Lines & Jardine (2019) [51]	Ndilo, Dettah	Community	Yellowknives Dene First Nation	15 Indigenous participants 13–18 years	Youth and researcher	Photovoice, mural art, sharing circles, observations, field notes, personal reflections	BROAD
MacDonald et al. (2015) [52]	Nunatisiavut	Community	Inuit	17 Indigenous participants 15–25 years	Youth	In-depth, semi-structured interviews	YES
McHugh et al. (2014) [53]	Alberta	Community	Métis, First Nation, Aboriginal	8 Indigenous participants All F 15–18 years	Youth	Semi-structured interviews	BROAD
Mikraszewicz & Richmond (2019) [54]	Biigtigong Nishnaabeg	Community	Anishinaabe	9 Indigenous participants (5 youth, 4 adults) Youth: 14–18 years	Youth, and community adults and Elders	Interviews	BROAD
Navia et al. (2018) [55]	Calgary	Community	NR	20 Indigenous participants 11F 9M 18–29 years	Retrospective youth	Interviews and art methods	BROAD
Nightingale & Richmond (2021) [69] Nightingale & Richmond (2022) [80]	Biigtigong & Mountain Lake Camp	Community	Anishinaabe	15 Indigenous participants (4 Elders/knowledge holders, 6 students, 5 camp staff) 11 Indigenous participants (6 students, 5 camp staff)	Youth, Elders/knowledge holders and community camp staff Youth, camp staff	Flexible interviews In-depth story-based interviews	BROAD
Oliver et al. (2020) [56]	Vancouver	Community & service delivery	NR	13 participants (4 Indigenous participants). 9F 4M	Foster parents (level of experience between <1–>20 years)	Semi-structured interviews	BROAD
Pace & Gabel (2018) [57]	St Lewis, Labrador	Community	Southern Inuit	10 Indigenous participants (5 youth, 5 older) Youth: 2F 3M; Adults: 5F 8–24 years: 5; 50–75 years: 5	Youth and older community members	Co-design workshops and online survey	BROAD
Parlee & O’Neil (2007) [58]	Lutsel K’e	Community	Chipewyan Dene	NR	Community members	Open-ended interviews	YES
Quinn (2012) [71]	Ontario	Community	NR	7 Indigenous participants 4F 3M 27–69 years	Retrospective youth proxy	Semi-structured interviews	BROAD
Ritchie et al. (2014) [59]	Ontario	Community	Wikwemikong Unceded Indian Reserve	43 Indigenous participants 16F 27M 12–19 years	Youth	Journals, interviews, talking circles and Elder teachings	YES
Sasakamoose et al. (2016) [60]	Canadian prairies	Community	First Nations and Métis	13 Indigenous participants 14–17 years	Youth	Sharing circles	YES
Shea et al. (2013) [61]	Battleford Tribal Council Region	Community	First Nations and Métis	Participant number NR All F 13–16 years	Youth	Photovoice, individual interviews, sharing circles, surveys	YES
Skinner & Masuda (2013) [62]	Winnipeg	Community	NR	8 Indigenous participants 13–20 years	Youth	Focus groups & rap, dance, poetry, photography, painting, mixed media	BROAD
Sloan Morgan, Thomas & McNab-Coombs (2022) [81]	Northern British Columbia	Community	First Nations	6 Indigenous participants	Youth	Photovoice	BROAD
Spiegel et al. (2020) [63]	British Columbia	Community	Tsleil-Waututh Nation	Limited description—a mix of family participants within the community	Youth, Elders and families	PhotoVoice & multiple discussion sessions with photos guiding discussions	BROAD
Tang & Jardine (2016) [72]	Northwest Canada	Community	Yellowknives Dene	30 Indigenous participants (11 community members, 19 children)	Youth, parents and community members	Participatory videos by youth & unstructured interviews (youth). Community focus groups (community members)	BROAD
Thompson et al. (2013) [64]	NR	Community	First Nations	15 Indigenous participants 14F 1M	Grandparents	Interview	YES
Victor et al. (2016) [65]	Sasketchewan	School setting	First Nations	14 participants (most identifying as Cree) Grade 8–11	Youth	Participatory visual photography; interviews; co-researching	YES
Wahi et al. (2020) [73]	Ontario & Alberta	Community	Ermineskin Cree Nation, Louis Bull Cree Nation, Samson Cree Nation, and Montana Cree Nation	60 Indigenous participants (current caregivers of children < 5 years, community members with Indigenous knowledge and community members providing health services)	Caregivers, Elders and community service providers	Single, face-to-face, one-to-one, in-depth, semi-structured interview	BROAD
Walls et al. (2014) [74]	Central Canada	Community	First Nations	66 Indigenous participants (30 Elders, 12 service providers) 21F 21M	Elders and service providers	Focus groups	BROAD
Walsh et al. (2020) [75]	Ontario	Community	Cree	3 Indigenous participants (involved with the land-based intervention the study was based off)	Service providers	Focus group	BROAD
Ward et al. (2021) [76]	Newfoundland & Labrador	Community	Innu	39 Indigenous participants 17–19 years (focus groups); 70+ years (interviews)	Youth and community members	Interviews and focus groups	YES
Yuen et al. (2013) [66]	Sasketchewan	School	Cree, Saulteaux, Dakota, Nakota, and Lakota	18 participants (not specified as Indigenous) 10F 8M Grade 7/8	Youth	Collaborative activities—games, arts	BROAD
**AUSTRALIA**							
Andersen et al. (2016) [83]	Western Sydney	Community	NR	38 participants (35 Indigenous) 22F 13M 3NR	Familial and service proxy (staff at Aboriginal medical service)	Focus groups	BROAD
Canuto et al. (2019) [84]	Yalata, Coober Pedy, Port Lincoln, Adelaide	Community	Aboriginal and/or Torres Strait Islander	46 Indigenous participants All M 18+ years	Male parents or caregivers	Yarning circle discussions	BROAD
Chamberlain et al. (2021) [85]	Melbourne, Alice Springs, Adelaide	Community	Aboriginal and Torres Strait Islander	17 Indigenous participants 15F 2M Mean age 29 years	Parents	Parent interviews and discussion groups	BROAD
Chenall & Senior (2009) [86]	Northern Territory	Community, school and clinic	Australian Indigenous	111 participants (not specified as Indigenous— 21 community-based informants; 22 high school students; 8 young women; 50 other community members; 20 non-Aboriginal community members) 42F 27M 42NR High school students: 13–19 years; other informants: <30–50+ years	Youth, community members, school teachers, clinic staff and council staff.	Discussions and workshops	YES
Clark et al. (2010) [82]	Tambellup	Community	Noongar	37 participants (23 Indigenous)	Aboriginal adults and non-Aboriginal leaders from community	Semi-structured interviews with both groups	BROAD
Crowe et al. (2017) [87]	South Coast New South Wales	Community and schools	Australian Indigenous	40 Indigenous participants 24F 16M 12–15 years	Youth	Interviews and surveys	BROAD
Dennison et al. (2014) [88]	Far North Queensland	Prison	Australian Indigenous	41 Indigenous participants All M 21–50 years	Indigenous fathers	Brief questionnaire and a semi-structured interview	BROAD
Gee et al. (2022) [89]	Victoria	Community	Koori	6 Indigenous participants 5F 1M 35–55 years.	Parents	Semi-structured tool and yarning circles	BROAD
Gibson et al. (2020) [90]	Wiradjuri country.	Community	Aboriginal	16 Indigenous participants	Elders	Yarning circle discussion	BROAD
Helmer et al. (2015) [91]	Western Australia, Northern Territory, South Australia	Community	NR	171 participants (88 Indigenous) 100F 71M 16–25 years	Youth	Group discussions and body mapping	BROAD
Johnston et al. (2007) [92]	Maningrida	Community	Maningrida Indigenous Australians	13 Indigenous participants 11F 2M 22–51 years	Adults in the community	Semi-structured interviews	BROAD
Kickett-Tucker (2009) [93]	Perth	Community and schools	Noongar	154 Indigenous participants (focus groups 120; interviews 34) Focus groups: 60F 60M; interviews: 18F 17M Focus groups: 13–17 years; interviews: 8–12 years	Youth	Focus groups and interviews	BROAD
Kiraly et al. (2015) [94]	Melbourne	Community	Indigenous Australian	430 participants (57 looking after Indigenous children; 15 Indigenous) 53F 2M 50–60 years	Caregivers and foster parents	Survey and focus groups	BROAD
Kruske et al. (2012) [95]	Northern Australia	Community	Aboriginal	15 Indigenous mother and baby pairings, plus associated family. All F Mothers: 15–29 years	Mothers, fathers and family members	Ethnographic; interviews every 4–6 weeks; photographs; field notes; observations	BROAD
Lowell et al. (2018) [96]	Northern Territory	Community	Yolŋu	36 Indigenous participants (30 community members, 6 children) Children: 3F 3M Children: 2mo–2 years; community members: 18–70 years	Family and community; researcher observations	Longitudinal case studies over 5 years with in-depth interviews, video-reflexive ethnography	BROAD
McCalman et al. (2020) [97]	Queensland	Boarding Schools	Aboriginal and Torres Strait Islander	9 participants (3 Indigenous) 6F 3M	Boarding school staff	Open-ended interview	BROAD
Miller et al. (2020) [98]	New South Wales	Community and health services	Aboriginal	425 participants (321 Indigenous) 383F 42M 18–50+ years	Parents and carers	Survey with open-ended questions	YES
Mohajer et al. (2009) [99]	Rural Australia	Community	Aboriginal	99 Indigenous participants 59F 40M 12–18 years	Youth	Individual interviews and/or focus group discussions	BROAD
Murrup-Stewart et al. (2021) [100]	Naarm/ Melbourne	Community	Aboriginal	20 Indigenous participants 14F 6M 18–27 years	Retrospective youth	One-on-one yarning sessions	YES
Povey et al. (2020) [101]	Northern Territory	Community	Aboriginal	45 Indigenous participants 10–18 years	Youth	Co-design workshops & online survey	YES
Priest, Mackean, et al. (2012) [102] Priest, Mackean, et al. (2012) [103]	Melbourne	Community; community-controlled health sector	Aboriginal	25 participants (not specified Indigenous) 18F 7M	Parents, family members, grandparents; and Aboriginal child or health workers; and foster parents	Interviews	YES
Priest et al. (2017) [104]	Melbourne	Community; community-controlled health sector	Koori	31 Indigenous participants 19F 12M 8–12 years	Youth	Focus groups and in-depth interviews	YES
Senior & Chenall (2012) [105]	Northern Territory	Community	Aboriginal	59 Indigenous participants All F 14–19 years	Youth	Focus groups	BROAD
Smith et al. (2020) [106]	Northern Territory	Community	Aboriginal and Torres Strait Islander	41 Indigenous participants (39 Yarning sessions; 18 individuals allowed social media access) All M 14–25 years	Youth	Yarning Sessions; Photovoice analysis of Facebook posts	BROAD
Williamson et al. (2010) [107]	Sydney	Community	Aboriginal	47 participants (not specified Indigenous) 30F 17M	Parents and Aboriginal health workers	Semi-structured focus groups and small-group interviews	YES
Young et al. (2017) [108]	New South Wales	Community controlled health services	Aboriginal	36 participants (not specified Indigenous) 24F 12M 18–65+ year	Community members, health service professionals and youth workers	Interviews	YES
**AOTEAROA NEW ZEALAND**							
Abel et al. (2001) [109]	Auckland	Community health service	Māori	150 participants (26 Māori; others Tongan, Samoan, Cook islands, Niuean, Pakeha) Māori: 17F 9M Mid-teens to early 40s	Parents or grandparents	Focus groups	BROAD
Abel et al. (2015) [110]	Hawkes Bay and Tairawhiti	Community	Māori	22 Māori participants (12 mothers of Māori infants, and 10 key informants) Mothers: 12F 19–39 years	Mothers	Focus groups	BROAD
Adcock et al. (2021) [111]	NR	Hospital	Māori	28 Māori participants (19 mothers, 5 fathers, 2 NICU peers, 1 aunt, 1 grandmother) 23F 5M	Family proxy	Focused life story interviews	BROAD
Beavis et al. (2019) [112]	Wellington	Community	Māori	18 Māori participants (11 children, 7 adults Tamariki/Rangatahi: 2–18 years; Adults: 22–43 years	Youth, caregivers and researchers	Adapted-ethnographic study	BROAD
Carlson et al. (2022) [113]	Tāmaki Makaurau (Auckland)	Community	Māori	22 Māori participants (total 56 participants) 16–20 years	Youth	Open-ended individual interviews	YES
Hamley et al. (2021) [114]	Aotearoa broadly	Community	Māori	23 Māori Rangatahi (27 other non-Māori participants) 34F 16M 1NR 12–22 years	Youth	Interviews	BROAD
Moewaka Barnes et al. (2019) [115]	Auckland	School	Māori	400 students (not specified Māori)	Youth, key informants	Survey with open-ended questions	BROAD
Page & Rona (2021) [116]	Te Ōnewanewa	Community	Māori	Rangatahi participants Other details not reported	Youth	Hui (meeting/gathering)	YES
**UNITED STATES**							
Ayunerak et al. (2014) [117]	Southwest Alaska	Community	Yup’ik	4 Indigenous participants	Community members and Elders	Narrative manuscript	BROAD
Bjorum (2014) [118]	Maine	Community	Wabanaki	11 participants (10 Indigenous) 9F 2M	Community members and child welfare staff	Focus groups; semi-structured, open-ended design	BROAD
Burnette & Cannon (2014) [119]	South-eastern USA	Community	South-eastern tribe	29 Indigenous participants All F 22–74 years	Mothers and female tribe members	Life history interviews; semi-structured	BROAD
Cross & Day (2008) [120]	NR	Community	American Indian	8 youth-grandparent Indigenous dyads Children: 4F 4M; Grandparents: 7F 1M. Children: 11–17 years; Grandparents: 51–72 years.	Youth and grandparents	Individual, in-person interviews	BROAD
Dalla et al. (2010) [121]	Navajo reservation	Community	Navajo	21 Indigenous participants All F 16–37 years	Young mothers and older mothers	Interviews	BROAD
de Schweinitz et. al.(2017) [122]	Alaska rural interior	Community	Athabascan	37 Indigenous participants 28F 9M	Youth and adults in the community	Focus groups	YES
DeCou et al. (2013) [123]	Alaska	Community	Alaska Native	25 Indigenous participants 18F 7M 18–37 years	Retrospective youth	Individual interviews	BROAD
Ford et al. (2012) [124]	Southwestern Alaska	Community	Yup’ik	25 Indigenous participants 11–18 years	Youth	Life history interviews	BROAD
Freeman (2019) [125]	Northern USA	Community	Rotinohshonni	19 Indigenous participants (14 youth, 5 adults) Youth: 11F 3M; Adults: 4F 1M	Youth and adults	Interviews	YES
Friesen et al. (2015) [126]	NR	Community	American Indian, Alaska Native	33 Indigenous participants 21F 12M 17–23 years	Youth and early adults	Interviews and focus groups	BROAD
Goodkind et al. (2012) [127]	Southwestern USA	Community reservation	Diné (Navajo)	37 Indigenous participants (14 youth, 15 parents/guardians, 8 granparents) Youth: 8F 6M; Parents: 12F 3M; Grandparents: 8F Youth: 12–17 years; Parents: 24–49 years; Grandparents: 54–90 years	Youth, parents and grandparents	Individual interviews	YES
Hand (2006) [128]	Northern USA	Community	Ojibwe	Poorly described sample—ethnographic interviews of an Ojibwe community	Elders and community members, child welfare personnel	Critical ethnography	BROAD
House et al. (2006) [129]	Southwestern USA	Community	Southwestern American Indian	24 Indigenous participants (10 youth, 6 parents, 9 Elders) 13–90 years	Youth, parents and Elders	Focus groups	BROAD
Isaacson et al. (2018) [130]	Northern Plains reservation	Community	Plains tribe	14 Indigenous participants (8 youth, 6 Elders) Youth: 7F 1M Youth: 13–17 years	Youth and Elders	Talking circles	YES
Lewis et al. (2018) [131]	Dillingham	Community	Yup’ik	20 Indigenous participants 14F 6M 46–95 years	Grandparents	Semi-structured interviews	BROAD
McKinley et al. (2020) [132]	South-eastern USA	Community	Indigenous	436 Indigenous participants across two tribal communities Youth: 11–23 years; Adults: 24–54 years; Elders: 55+ years	Youth and community members	Individual interviews; family interviews; focus groups	YES
Nu & Bersamin (2017) [133]	Southwestern Alaska	Community	Yup’ik	Poor description of participants—community based study	Youth and community	Focus groups	BROAD
Rasmus et al. (2014) [134]	Bering Sea Coast Alaska	Community	Yup’ik	25 Indigenous participants 12F 13M 11–18 years	Youth	Interviews; life history & ‘memoing’ of interviews	YES
Strickland et al. (2006) [135]	Pacific Northwest	Community	Pacific Northwest Tribe	49 Indigenous participants (40 parents, 9 Elders)	Parents and Elders	Interviews and focus groups	BROAD
Trinidad (2009) [136]	Hawaii	Community	Native Hawaiian	17 participants (16 Indigenous—8 young adults, 4 youth staff, 2 parents, 2 board members, 1 Elder) 17–25 years youth	Youth, parents, Elders, community advocates	Open-ended interviews	BROAD
Trout et al. (2018) [137]	Alaska	Community	Inupiaq	17 youth researchers (11 Indigenous—10 adults in focus groups, 20 interviews with local researchers 14–25 years youth researchers	Youth, adults and Elders	Q&A sessions, photovoice, digital storytelling, interviews	BROAD
West et al. (2012) [138]	Chicago	Community	Chicago American Indian	107 Indigenous youth and families (15 youth participants) 71F 36M Youth: <18 years	Youth, family members and Elders	Focus groups	BROAD
Wexler (2006) [139] Wexler (2009) [140]	Northwest Alaska	Community	Inupiat	12 focus groups of 3–12 Indigenous participants >50% F 13–21 years	Youth	Focus groups	YES
Wexler (2013) [141]	Northwest Alaska	Community	Inupiaq	23 Indigenous participants (9 youth, 7 adults, 7 Elders) Youth: 14–21 years; Adults: 35–50 years; Elders: 60+ years.	Youth, adults and Elders	Focus groups and interviews; digital stories	BROAD
Wexler et al. (2013) [142] Wexler et al. (2014) [143]	Northwest Alaska	Community	Inupiaq	20 Indigenous participants 10F 10M 11–18 years	Youth	Interviews (3 x 1 h for each participant)	BROAD
Wood et al. (2018) [144]	San Diego	Community	Kumeyaay Luiseno	22 Indigenous participants 17F 5M 14–27 years	Youth and retrospective youth	In depth and semi-structured interviews; focus groups; surveys	YES

NR = not reported. Participant number, Indigenous number, split by ages, age range, gender are reported where available. 2SLGBTTQQIA = Two Spirit, lesbian, gay, bisexual, transgender, queer, questioning, intersex and allies. Two-spirit = Two-Spirit is used by and for Indigenous people as a way to relate to ourselves, our communities, and our spirits outside of a western colonial context; some people identify as having a spiritual balance between feminine and masculine energies [68].

##### Basic Resources for Survival

For many Indigenous youth in Canada, having access to the basic resources for survival is a common challenge to their wellbeing [41,44,45,49,54,57,58,62,63]. The condition of the land and environment [54,57,58,62], together with youths’ social and living conditions [44,45,67,71], underpin access to these resources. For Indigenous youth, access to basic resources significantly influences their capacity to maintain meaningful connections with others, which significantly affects wellbeing [41]. Challenges in securing money, housing and food are posed by mobility between urban locations and reserves [41,49]. Negative incursions into communities, such as mining, impact youths’ wellbeing in complex ways [63], with increased infrastructure and opportunity gained at the expense of water and food quality [58]. For younger Indigenous youth, wellbeing is understood largely within their care context [44,45]. Pervasive poverty can compel caregivers to focus on children’s survival rather than their thriving, limiting children’s prospects and compromising their wellbeing [44,45,49].

##### Safety and Stability

The importance of having a safe and stable living environment is essential to achieving wellbeing for Indigenous youth in Canada [40,41,43,44,45,46,47,48,49,52,53,54,55,56,58,60,61,62,63,64,65,66,67,70]. A number of negative incursions undermine the attainment of this safe environment: colonisation and racism [40,43,44,45,46,47,49,53,54,55,56,58,60,62,63,64,65,66,67,68,81] effects include microaggressions, marginalisation, violence, suicide and substance abuse [45,47,49,53,62,67,68,70,71,74,75,81]; experiences with the child welfare system that disrupt stable living [55,56,62,71,81]; and substance use and risky behaviours [45,46,60,61,64,66,67,81] that contribute to dangerous environments. Connections to land offer a stabilising influence, via opportunities to engage in cultural activities [52,54,58,79,80]. These connections can be disrupted by industry, pollution and experiences of upheaval, mobility and separation [63,64,66]. Colonising systems, such as child welfare, can alienate and disempower youth, separating them from vital connections with family, community and culture [41,55,56,68,71,80]. Indigenous youth are often cognisant of the damage that substance use, both their own and by their caregivers, has on their sense of safety, stability and wellbeing [45,46,49,67,70,74].

##### Relationships with Others

Relationships with others are central to the wellbeing of Indigenous youth in Canada, impacting their identity, resilience and outlook on life [41,42,43,45,46,47,48,49,51,52,53,54,55,56,57,58,59,60,61,62,63,64,65,66,68,79,80,81]. These relationships invoke a sense of belonging, tethering youth to their culture and identity [45,48,49,51,53,54,55,56,57,58,60,61,63,64,65,68,75,79,80,81] and are strengthened by engaging with traditional culture and lands [42,43,48,51,52,54,59,63,64,66,69,75,79,80]. This can be difficult in urban settings and in circumstances of mobility and transience [41,43,62,74]. Relationships with parents and caregivers provide guidance, support, and cultural knowledge which supports youth wellbeing [45,46,47,52,60,61,64,65,68,79,80,81]. Nurturing of children by caregivers can temper and challenge intergenerational trauma [47,49,55]. For some female Indigenous youth, motherhood offers an opportunity to forge new and unique relationships with their children that contribute positively to wellbeing [41,53]. Relationships with friends, peers and romantic partners are central to the wellbeing of youth [46,47,53,61,70], however, these can be complicated by substance use, peer pressure, suicide and societal forces [46,47,49,53,61,70,74].

##### Culture and Spirituality

Culture and spirituality are inextricable components of the wellbeing of Indigenous youth in Canada, which includes traditional language, knowledge, activities, beliefs and land [40,41,42,43,44,45,46,47,48,49,50,51,52,53,54,55,56,57,58,59,60,61,63,64,65,66,67,69,70,73,75,79,80,81]. The challenges and opportunities for youth living across ‘two worlds’—Indigenous and Western—enhances the wellbeing of some, while posing difficulties and uncomfortable trade-offs for others [40,41,47,48,52,57,60,64].

Maintaining traditional languages [42,43,48,56,61,64,79] and participating in cultural activities, such as subsistence living, sweats, traditional gatherings and talking circles [43,44,49,50,51,52,53,54,57,59,60,63,64,65,66,69,70,75,76,79,80], are sources of cultural strength, physical and mental health and wellbeing, and are particularly important for youth who have been disconnected from family [44,55,56,70,71,80]. These activities are critical for the transmission of cultural knowledge and wellbeing to the next generation [48,51,54,57,58,63,64,65,66,69,73,75,76,79,80,81]. Spirituality, which is closely associated with traditional cultural practices [45,54,59,60,63,64,70,71,75,80] and nature and land [53,54,56,63,64,71,75,79,80], is a source of strength, resilience and identity for Indigenous youth [59,60,61,63,64,70,71,75,80,81]. Western religion is present in some Indigenous youths’ lives but is sometimes negatively associated with colonization [64]. Intergenerational trauma and contemporary child welfare practices erode cultural connections, however many Indigenous youth are striving to reconnect with traditional culture [45,55,56,64,71,80,81]. The importance of mental and physical health to wellbeing are commonly understood by Indigenous youth within cultural bounds [46,51,52,54,59,60,63,71,73] and holistic frameworks, such as the Medicine Wheel [46].

Land is a key component of cultural connection, and a medium through which culture is experienced, practiced and continued [42,51,52,54,63,64,69,79]. Connection with land is, however, complicated by transiency and a common disconnect between urban and rural settings [40,41,42,43,48,49,51,52,54,56,57,58,59,60,62,63,64,65,67]. City living is often associated with criminalisation, discrimination and substance use [45,49,52,62], while spending time on traditional territories enables engagement with cultural activities, furthers youth perception of their culture, history, sense of self and their place within their world [51,52,54,59,64,65,69,79]. Environmental deterioration and climate change disrupts Indigenous youths’ connections to land and culture, as well as damaging health, food, economic opportunities and living conditions, which all impact deleteriously on wellbeing [51,52,54,57,58,60,62,63].

##### Knowledge, Opportunities and the Future

Indigenous youth in Canada grapple to balance traditional customs, values and priorities with the demands of a challenging and uncertain future [40,45,46,48,49,51,52,54,55,56,57,58,60,61,63,64,66,79]. Despite culture’s centrality to Indigenous youths’ wellbeing, maintaining culture while also engaging with mainstream education, employment and expectations is challenging [45,60]. Uncertainty, particularly stemming from environmental degradation [52,57,58,61,63], experiences of violence, suicide and peer pressure [45,46,47], undermine youth’s future aspirations [55]. Knowledge about opportunities and youth being able to control or contribute to associated programs, can greatly impact on Indigenous youths’ outlook on life and the future [40,41,53]. With greater access to services, education and employment opportunities in urban locations [49], the choice between remaining on traditional lands or moving is difficult for many Indigenous youths [40,74]. Caregivers feel the need to support youth to ensure they are well resourced to have opportunities, while still fostering their cultural connections [58,60,64]. While cultural responsibilities are important to wellbeing [52], they can feel restrictive and stifling for some youth [40,57]. When knowledge, educational and vocational opportunities for Indigenous youth are grounded in traditional culture, this cultivates pride and achievement, increasing wellbeing [46,48,49,51,53,54,56,57,60,61,64,66,80].

##### Identity

Wellbeing among Indigenous youth in Canada is strongly associated with identity and resilience [40,41,42,43,45,49,51,53,54,55,56,58,59,62,64,65,66,68,70,79]. While colonisation has undermined many parts of life for Indigenous peoples in Canada, Indigenous youth remain cognisant of the value and strengths of their traditional culture, which can serve to protect and promote wellbeing [40,42,53,70,76,79]. Stereotypes in the media, experiences of racism and negative experiences within communities can cause identity dissonance for Indigenous youth; whereas, increased knowledge about Indigenous history and colonisation can provide youth with a greater appreciation of their own culture, strengthening self-esteem and wellbeing [40,42,49,54,58,62,64,66,79]. The need for youth to feel like they *belong* is key to wellbeing: [41,62,64,70,79] knowing who you are and where you come from underpins this sense of belonging, bestowing a sense of place and identity [42,43,45,53,58,59,64,68,76]. Indigenous youth living in urban settings develop their own distinctive identity that melds their Indigenous culture to that of the urban cultural setting they live in [55,62]. Participation in cultural activities, including dancing, art, smudging and sweats, is important for building a sense of belonging, as well as fostering feelings of strength, pride, identity and wellbeing [45,51,55,56,58,59,65,66,70,76,79].

##### Resilience and Independence

Many Indigenous youth in Canada experience a range of challenges in their lives, which require strength and resistance to overcome and to maintain and improve their wellbeing [50,52,55,58,60,61,63,65,70,79]. While these negative experiences can undermine wellbeing, sense of agency and inflict pain [50], challenges in early life can build resilience, strength and pride [44,57]. Resistance against stereotypes and subjugation, expressed via culture, art and trusted community connections, affords young people strength and bolsters their identity [52,65,70,79], whilst acknowledgement of issues faced facilitates forward momentum [51]. The inclusion of youth in community decision making and program development is also seen as important for their independence, as they are experts in their own lives [49,51,58]. In the face of a changing world, impacted by Western forces and climate change, youth find resilience in culture [52]. Many Indigenous youth assume responsibilities for the care of themselves and others while they are still relatively young [41,60]. Caring for others, including siblings and their own children, is seen as an opportunity to reclaim their autonomy, break the cycle of trauma, maintain cultural continuity and build independence [52,55,61,70].

##### Recreation and Interests

Engaging in recreation and having interests was described as important to the wellbeing of Indigenous youth in Canada via reducing stress, improving health, having fun and connecting young people in a positive way [48,49,51,52,58,60,61,65,66,79]. Recreation includes sports, educational activities, art and cultural activities [48,60,79,80]. with environmental changes sometimes challenging access to these activities [49,52]. Participating in sport promotes happiness and health for Indigenous youth [49,51]. Moreover, sport can help young people who have experienced trauma and/or separation from family and community create a new identity, take part in community and develop aspirations for the future [60,66]. Engaging in physical activities, including cultural activities like fishing and hunting and structured sports, helps Indigenous youth to focus on positive things, to achieve better at school, and to avoid risky behaviours [60,66,79]. Avoiding stereotypes, particularly for girls, and focusing on having fun was put forward as important for wellbeing [61]. Participation in arts-based activities facilitates Indigenous youths’ self-knowledge via self-expression, which can nurture cultural identity and help youth to express important issues and relationships [65].

#### 3.2.2. Indigenous Youth in the USA

Three Indigenous groups reside in the USA: American Indian, Alaska Native (AI/AN) and Native Hawaiian youth. The Indigenous population in the USA is diverse: there are 574 unique federally recognised American Indian Tribes, each with their own culture. AI/AN communities include Indian Reservation, Pueblos, Villages in Alaska, and other places set aside for AI/AN peoples. These communities and places are often referred to collectively as Indian Reservations or Indian Country. Such places can be on traditional lands or lands, which were not originally traditional for the many Tribes who were forcefully relocated. Altogether, they are Indigenous lands. Native Hawaiian peoples have occupied the Hawaiian archipelago under various political and regal structures since approximately 1000–1200 CE. Colonial influences from the USA have been present in Hawaii since the 1800s, with Hawaii incorporated as state of the USA in 1959 [145]. The influence of colonisation on Native Hawaiian peoples has wrought many of the same harmful effects experienced by other Indigenous peoples in CANZUS nations [146]. Indigenous peoples living in the USA continue to navigate governmental impacts on their lives, advocating for justice across the issues of child welfare, jurisdiction, protection of land and nature, sovereignty and, in some cases, reparations for historical colonial impacts on their lives [147,148].

Twenty-eight studies in this review reported on the wellbeing of Indigenous youth in the USA [117,118,119,120,121,122,123,124,125,126,127,128,129,130,131,132,133,134,135,136,137,138,139,140,141,142,143,144]. Our thematic analysis identified six overarching components of wellbeing for this population: safety and basic needs; relationships and connection; cultural identity and pride; looking to the past and the future; and being healthy. These domains reveal the enduring importance of connection with others as a way to anchor Indigenous youth in the USA to their culture and community and to reveal a path for Indigenous youth into an uncertain future.

The current review includes only one paper with perspectives from Native Hawaiian youth, which has been referenced where findings apply to Native Hawaiian youth [136]. We are unlikely to have captured the experience of wellbeing for young Native Hawaiians in this review.

##### Safety and Basic Needs

The wellbeing of Indigenous youth in the USA is influenced by their access to a safe environment and basic resources for living, the nature of which differs for youth living on Native Reservations and those living in urban environments [118,119,120,121,122,124,126,127,128,134,135,136,137,138,139,140,141,143,144]. Indigenous youth who live in isolated locations, including Native Reservations, sometimes experience a greater sense of safety and stability, however these benefits can be tempered by poor access to utilities (water and electricity) and experiences of colonial-related intrusions like violence and substance abuse [121,144]. Complex family situations, involving dislocated and broken families, and sometimes violence, negatively impact on youth wellbeing [119,121], leading Indigenous youth to seek out safety with supportive friends and other family [134,140,143]. The impact of colonial pressures has resulted in some youth experiencing violence, alcohol abuse and illicit drug use, which can serve to reinforce cycles of trauma and disadvantage [120,121,126,127,128,135,136,137,138,139]. Youth programs and support services can foster youth wellbeing by offering stable environments and supporting connection to culture [118,126].

##### Relationships and Connection

Relationships for Indigenous youth in the USA are central to their wellbeing. Connections with community, family, Elders and peers are key contributing factors to how Indigenous youth develop a sense of self and feelings of belonging [117,118,119,120,121,122,123,124,125,127,128,129,130,132,133,134,135,136,137,138,139,140,141,142,143,144]. Feeling part of a community, for youth, is fostered through engagement with traditional cultural activities that instil pride and identity [117,120,123,125,129,132,133,134,135,137,138,139,140,143,144]. Participation as a community member is often centrally important in developing Indigenous youths’ identity [124,133,139,142,143] and resilience [127,142,143]. In circumstances where youth are forcibly removed from family and community settings, there is often damage to important relationships, connections and opportunities to learn and share culture [123,127,128,129,130,137,143,144]. Violence and substance abuse, resulting from colonial influences and intergenerational trauma, weigh negatively on youth and their communities [119,120,121,122,134,135,136,138,139,141,144], and youth are sometimes faced with difficult decisions to remain with families or sever these central relationships to avoid such behaviours [135,140,141]. Peer connections can provide support for Indigenous youth when dealing with challenging family relationships [127,130,139,142,143], however, peer interactions can sometimes also be settings orf violence, bullying, substance abuse and mental health issues [120,121,134,138,140,142]. Positive role models are important for Indigenous youths’ perceptions of their future [130,134,140,142,143].

##### Culture and Tradition

The wellbeing of Indigenous youth in the USA is connected closely to traditional culture and practices, which are under constant pressure from colonialism and racism [117,118,122,123,124,125,126,127,128,129,130,131,132,133,134,135,136,137,138,139,140,141,142,143,144]. Traditional community-based activities, such as subsistence based living (including hunting and fishing), and cultural practices, such as smudging and pow-wows, provide youth with a sense of purpose, connection and cultural pride [117,118,123,128,129,130,131,132,133,134,137,143,144]. The passing on of language closely bonds Indigenous generations [122,125,127,137,138,141], however, there are a decreasing number of fluent native language speakers to ensure continuation through the next generation [117,126,127,129,137]. Opportunities to learn traditional language and culture through the mainstream education system are not often available to youth, however, integrating traditional and mainstream knowledge is beneficial in supporting the identity and wellbeing of Indigenous youth [130,137].

On Indian Reservations, land and animals, such as horses, are key components of youths’ experience of wellbeing, as they facilitate and deepen cultural connections to traditional activities, spirit and ancestors [122,123,125,130,136,137,143,144]. This physical connection to traditional lands is challenged by the demands of living in ‘two-worlds’—the traditional and the contemporary mainstream [117,123,127,136,137]. Remaining on traditional lands fosters cultural connections and improved youth wellbeing, however, this can be associated with boredom and reduced opportunities for education, employment and financial stability due to the often remote locations [137,139,143]. Traditional Indigenous environments have been permeated by pressures and problems resulting from colonialisation, religiosity and the loss of traditional culture [136,137,138]. This is evidenced by instances of suicide, self-harm, violence and substance abuse experienced by Indigenous youth living on Indian Reservations and within traditional communities [117,119,122,137,139]. Indigenous youth, with support from Elders and their communities, use traditional practices, spirituality and culture, to foster wellbeing in the face of the challenges caused by the ongoing effects of colonisation and intergenerational trauma [117,118,122,123,132].

##### Cultural Identity and Pride

A common theme was the importance of Indigenous youth in the USA having a strong and positive self-identity to strengthen their wellbeing [117,118,121,122,124,125,126,129,136,137,138,139,140,142,143,144]. The identity of many Indigenous youth is grounded in culture, tradition and their relationships with others, which supports and is supported by the experience of hope and capacity for choice. The identities of Indigenous youth, however, are multifaceted and complex, and this young population faces increasing challenges to preserving the strength of their traditional identity [117,118,121,122,124,125,126,129,136,137,138,139,140,142,143,144].

Indigenous youth who have strong, supportive family (including extended families), community ties and knowledge of their cultural roots, have a more unified identity [117,118]. This provides a sense of belonging, stability and an understanding of their place in the world [117,118,138]. Land and place is particularly important to the identity of Indigenous youth in delineating their relationships with other people, the natural and spiritual worlds, and with the past and future [125,136]. Colonisation, war, boarding schools, and associated trauma has wrought great damage on Indigenous peoples, with deleterious effects on wellbeing a result of such impacts [138,144]. This has brought catastrophic mental health issues for Indigenous youth, including anxiety, depression and suicide [138]. It is clear, however, that many Indigenous youths are determined to reclaim their cultural strengths and restore pride in their Native identity, via learning traditional languages and stories, and engaging in subsistence-related activities [117,118,125,126,136,137,138,142,143,144]. Working to ‘give back’ to their communities is a way in which Indigenous youth cultivate and strengthen their identity and instils feelings of acceptance and belonging, as well as helping others to feel pride in their Native identity [144].

##### Looking to the Past and the Future

Indigenous youth in the USA experience tension as they try to negotiate a balance between traditional Indigenous culture, values and practices with the profound pressures of an uncertain future. Having a purpose and plan for the future is important to Indigenous youth wellbeing [117,118,120,121,122,123,124,126,127,128,130,131,132,135,136,137,138,139,140,141,142,143,144], however, Indigenous youths’ aspirations are circumscribed by their experiences and environment [126,127]. Some Indigenous youth feel lost, without direction, and without a sense that they have a future purpose, regardless of educational attainment [139,142]. Parents and communities sometimes attribute this lack of purpose to the degradation of traditional cultural roles and influx of technology [117,139]. This is especially true for males who, without traditional subsistence activities, may be unsure of how to contribute the larger community [139,140]. Youth purposelessness within communities is related to the negative impacts of colonisation that results in subsequent destructive community incursions like suicide and substance abuse [139,140,142,144]. Whilst living in traditional communities tends to improve youth wellbeing through gaining a sense of responsibility, belonging and understanding of traditional knowledge [123], this embedding of cultural life can be restrictive and disheartening, due to inequitable structuring of education and employment opportunities that impact negatively on wellbeing [138,140,144]. Urban environments can be stifling and alienating also, due to absence of cultural comforts, despite offering more opportunity to youth [138,142,144]. The limited employment opportunities available to many Indigenous youth results in financial strain [121,126,140,141] and poor mental health [122,138].

While mainstream education is regarded as important for future employment for Indigenous youths, it is also associated with deeply adverse experiences for their communities [135,139], and often necessitates the dislocation of youths from their culture and supportive relationships [130,131,141]. Employment is sometimes seen as a means of contributing positively to the community [135], however, such opportunities are not always available, particularly in remote locations, and this responsibility can weigh heavily on youth [140,142].

Survival and resilience, both in the youth themselves and their broader community and culture, are important contributors to youths’ future hopes and wellbeing [137]. A key aspect of survival is Indigenous youths’ ability to adapt to change in their lives, within Western spaces and in response to modern challenges [136,137]. Such resilience is fostered by cultural traditions [123,125,132,137,138], strong interpersonal relationships [138,139], and an understanding of how previous generations survived colonial histories and racism in the present and past [127,139,143,144]. Caregivers prioritise passing down morals and values to youth in an act of cultural continuity, which was found to arm them with future purpose and a resilient sense of their own wellbeing [122,123,127,137,144].

##### Being Healthy

Being healthy was spoken about as being important to wellbeing for Indigenous youth in the USA, which encompassed a holistic understanding of physical, mental and spiritual health through balance [120,121,122,123,124,126,127,132,136,138,139,140,141,142,143,144]. The deleterious effects of colonisation on the health and wellbeing of Indigenous peoples, including high rates of chronic diseases, drug and alcohol abuse, and suicide, occasion great sadness, anger and grief for Indigenous youth [127,136,139,140,141,142,143]. Indigenous youth regard living on Reservations and participation in subsistence activities as critical to improving their health and wellbeing. The health benefits of exercise, traditional diets, and the psychological benefits associated with engaging with nature and working together with others, strengthens relational and cultural connections and improves balance of all aspects of holistic health [123,139,140,141,142,143,144]. Participation in sports and healthy diet programs were also described as important for supporting the health of Indigenous youth [138].

#### 3.2.3. Māori Youth in Aotearoa New Zealand

Māori are descendants of Polynesian peoples who arrived in Aotearoa New Zealand around 1300 CE, and are known as *tangata whenua* (the people of the land) [149]. Since European contact in the 1700s, Māori have experienced periods of conflict, land appropriation and marginalisation within their own lands [150]. More recently, Māori calls for self-determination, and advocacy for *te reo* Māori (the Māori language), *tikanga* Māori (ways of being), and *mātauranga* Māori (Māori knowledge) have expanded how wellbeing is conceptualised within the health system in Aotearoa New Zealand.

There were eight papers that reported aspects of wellbeing of Māori *rangatahi* (youth, 12–25 years) and *tamariki* (children, 0–14 years) in Aotearoa New Zealand [109,110,111,112,113,114,115,116]. We use the term *rangatahi* to reference older youth (in this paper, this is up to 18 years), and *tamariki* where results are specific to young Māori children. The wellbeing of *rangatahi* and *tamariki* is split into six overarching components: belonging, care and support; culture; knowledge and knowing; identity and agency; and physical health.

Due to the systematic nature of this review and the need for consistency across countries, only peer-reviewed journals were searched to obtain papers from Aotearoa New Zealand. As much research in Aotearoa New Zealand sits outside traditional Western journals, it is unlikely that the current review has captured the breadth of literature reporting on the wellbeing of *rangatahi* and *tamariki* that can be found in community journals and grey literature.

##### Belonging, Care and Support

Feeling supported and cared for is a key aspect of *hauora* (wellbeing), for *rangatahi* and *tamariki*, which can be moderated by their sense of belonging [109,110,111,112,113,114,115,116]. *Whānau* (a wider conceptualisation of family that also includes extended family members and friends) [116], community, and health professional support established during infancy is regarded as key to *tamariki* wellbeing [109,110,114,116]. This support begins very early in life, and is reinforced through cultural items, such as the *wahakura* (flax bassinet), which grounds *tamariki* to the *whenua* (land) and to a sense of safety and belonging to their culture [110]. Physical closeness and breastfeeding are valued in enabling bonding between whānau, parents and the child [109,110,111,116]. Caregivers prioritise *rangatahi* and *tamariki* physical health, even in the face of pressures like food insecurity [112] and pre-term births [111]. *Kaumātua* (grandparents, elders) and *pakeke* (adults) play important roles in nurturing *hauora* [114], and this is further reinforced in relationships with whanau members such as siblings, who provide important aspects of cultural connection for *rangatahi* and *tamariki* [111,114,116]. The concept of whānau is collective and ties into many complex aspects of *hauora* for *tamariki* and *rangatahi*: there is support and connection realised in relationships with whānau and *pakeke* [113,114]; and sadness is seen when deaths and mental illness are present in the community [113,116]. *Whanaungatanga* (nurturing of relationships) is inextricably tied to *hauora* and the concept of *whakapapa* (genealogy) [114]. A sense of belonging within whānau and *te Ao Māori* (the Māori world) is crucial in maintaining and strengthening connection to each other, to *whakapapa*, and to the *whenua*. This is further exemplified by the concept of *kaitiakitanga* (stewardship, guardianship) which demonstrates the significance of reciprocity and belonging experienced in relation to land and people [116]. As mentioned, Māori are known as *tangata whenua*, people of the land, with the relationship between *whenua* and *tāngata* (people) intrinsically important in *hauora*. These relationships facilitate the development of an understanding of self that is not as an individual, but part of a wider collective, and thus all aspects of these relational spaces contribute to the *hauora* of *rangatahi* [111,112,114,115,116].

##### Ahurea (Culture)

Culture is a significant aspect of *hauora* for *rangatahi* and *tamariki* [109,110,111,112,113,114,115,116]. Cultural practices are vital in the provision of traditional knowledge, cultural grounding and connection to *tīpuna* (ancestors). Cultural practices during early life, such as burying the *whenua* (placenta) in the *whenua* [109,111], being given a Māori name [109,111], and being placed in *wahakura* [110] connect *tamariki* to their *tīpuna*, their *whenua*, and imbues strength and spiritual protection [109,110,111]. These actions place *tamariki* and *rangatahi* Māori within the housing of whānau *Māori*, which encircles them within the past, present, and future [114,116]. Connections to *tīpuna* can also come through physical visitations to significant land sites, who often are personifications and embodiments of *tīpuna* themselves, carrying significant cultural value for Māori [116]. *Whakawhanaungatanga* (process of establishing relationships, relating well to others) is fostered for *tamariki* and *rangatahi* through these cultural practices [113]. *Tamariki* are crucial for the continuation of *whakapapa* within their whānau and *iwi*—therefore, then nurturing of *tamariki* by whānau is critically important in early life [111,114]. Relationships with whānau conceptualised within Māori understandings of time and space can strengthen cultural understandings, whilst reciprocally strengthening *whakapapa* and *hauora* [114]. Additionally, connections to *whenua*, through learned practices such as *mahi māra* (gardening), *waka ama* (traditional outrigger canoeing), *kapa haka* (Māori performing arts), *kaitiakitanga* and *te reo Māori* [114,116], allow *tamariki* and *rangatahi* to foster connections with whānau and *te Ao Māori*, further enhancing *hauora* [112,114,116].

##### *Mātauranga Māori* (Māori Knowledge) and *Mōhiotanga* (Knowing)

The transmission of Māori knowledge and access to education is an essential foundation of *rangatahi* and *tamariki* wellbeing. Within *whānau*, food-based knowledge systems that iterate the importance of sustainable practice and reciprocity are seen as a practical solution to facilitating positive *hauora* outcomes throughout the lifetime [112]. Incorporating *mātauranga Māori* into formal education settings enables *tamariki* and *rangatahi* to understand education and occupational opportunities that exist in the future, including options that privilege *Kaupapa Māori* (a way of doing things from a Māori worldview) and connect with all aspects of *te Ao Māori*, such as the *taiao* (environment), instead of strictly Western-style opportunities that may not foster their worldview [114,115]. Maintaining *Kaupapa Māori* in education, and creation and teaching of knowledge, contributes positively to the health and wellbeing of *rangatahi* and *tamariki* [115].

##### Identity and Agency

A strong understanding of one’s identity and sense of agency is intrinsic to the *hauora* of *rangatahi* and *tamariki*. These important contributing factors to *hauora* build both a sense of independence and also of interdependence through the strengthening of connection with their whanau [109,113,115]. Some Māori families place particular value on fostering the independence of *tamariki* from a young age, specifically in the context of independence in sleeping situations [109]. Other perspectives emphasise the interdependent and collective nature of the whānau, with *tamariki* and *rangatahi* being intrinsically ensconced and supported within this collective, giving rise to the identity of *tamariki* and *rangatahi* within the context of whānau *Māori* [113,114,116]. Cultural associations, such as parents choosing Māori names [109,111] and *tamariki* having the knowledge to share their life narratives, instils a strong sense of identity for *tamariki* and *rangatahi* [109]. *Rangatahi* and *tamariki* agency and identity is strengthened through grounding knowledge within Māori paradigms, as well as within the natural world and spaces in communities, contributing to *hauora* by allowing *tamariki* and *rangatahi* to understand their place in the world [113,114,115]. These strong, culturally grounded pursuits are considered critical to the wellbeing of *tamariki* and *rangatahi*. The incursion of racism resulting from colonial pressures can have profound impacts on *rangatahi* and *tamariki,* complicating the positive effects of cultural identity on *hauora* [113].

##### Physical Health

Physical health is a key aspect of *rangatahi* and *tamariki hauora*. Access and availability of healthy foods is seen as important for good physical health for *rangatahi* and *tamariki* [112]. *Whānau* doing their best to provide such food, even in the face of food insecurity, is crucial in providing *rangatahi* and *tamariki* with a healthy foundation for their adult lives [112]. Physical activity is often seen in the context of *whānau kaupapa*, with reciprocal time spent together in nature or the local community beneficial for *rangatahi hauora* [114].

#### 3.2.4. Indigenous Youth in Australia

Indigenous peoples in Australia are comprised of Aboriginal and Torres Strait Islander peoples, representing the oldest continuing culture in the world. Aboriginal peoples are native to Australia, encompassing more than 250 unique languages and distinct tribal groups, holding strong ties to cultural lands known as ‘Country’ across Australia [151]. Torres Strait Islander peoples are native to the islands of the Torres Strait, a small cluster of islands off the north coast of Queensland in Australia, and like Aboriginal peoples, they too embody heterogenous cultural groups and live across the lands of Australia and the Torres Strait [151]. Both Aboriginal and Torres Strait Islander peoples are the First Peoples of Australia. Indigenous Australians continue to advocate for improved representation across Australian society and parliamentary structures, access to cultural lands, and self-determination [152].

There were 29 studies that reported on the wellbeing of Indigenous youth in Australia [82,83,84,85,86,87,88,89,90,91,92,93,94,95,96,97,98,99,100,101,102,103,104,105,106,107,108]. Our thematic analysis identified seven overarching components of wellbeing for this population: basic needs; relationships; culture; aspirations for the future; identity; recreational activities and interests; and physical and mental health.

These domains reveal the importance of connection with others within communities to foster strong relationships, strengthen youths’ identity, and ensure continuity of Indigenous knowledge.

##### Basic Needs

The provision of basic material needs and services is seen as an essential foundation upon which positive wellbeing can be realised for Indigenous youth in Australia [96,98]. Safe and stable housing is viewed as a key protective element against potential negative influences such as food insecurity, unsafe environments, transience, and exposure to communicable disease and the child welfare system [82,83,89,98,103,106,108]. Accommodation security can be undermined by unaffordable housing, discrimination when entering the rental market, and overcrowding, affecting Indigenous youths’ experience of safe housing [83,96,101].

Fresh and healthy food availability positively contributes to health and wellbeing for Indigenous youth in maintaining health [87,104]. Parents often strive to provide this as a key element of wellbeing for their children [89,98,103], which can be challenging due to housing instability, financial strain and the high price of food in remote communities [83,96,103]. Hunting, fishing and sharing food among the community is seen to enhance the wellbeing of all Indigenous people, including youth, in terms of food security, and cultural and community connections [106].

Parents’ and carers’ access to employment and other essential services is seen to contribute to wellbeing for Indigenous youth. This can be challenging in remote communities [99,106], as disconnection in parent-youth relationships may eventuate when parents and caregivers relocate for financial stability [84,106]. Tight financial situations can prevent young people from accessing basic needs and extra opportunities, such as sport [102]. Accessing health services can be easier for youth through community-controlled health clinics [82,85,98,101,106,107], however service providers report financial instability remains a barrier to access [97], and youth may be reluctant to access community clinics due to concerns around their privacy in small communities [99].

##### Relationships

Relationships within communities and kinship groups are critical to providing support, guidance, cultural mentorship, and role modelling for Indigenous youth in Australia [82,84,87,98,102,103,106], especially when parents are unable to be primary carers of their children [94,96,102]. Kinship relationships are highly valued in supporting young people to navigate two worlds: relationships with Elders provide Indigenous youth with cultural knowledge and are strengthened via participation in cultural activities [87,102,103]. Early exposure to such cultural activities has powerful impacts on establishing identity and wellbeing for Indigenous youth [87,107], fostering resilience and ensuring the continuation of cultural knowledge [82,88,103,108].

Where the immediate family can provide foundational basic needs such as a home, food, financial support, and love and care, Indigenous youths’ wellbeing is more strongly supported [98]. The influence of colonial structures, such as child welfare, can disrupt this supportive environment [103]. Families are sometimes seen as strict [105], and some parents express difficulties navigating the balance between overprotectiveness and freedom [89], which may result in limits on youths’ self-determination. Parents identify that working through their own personal trauma and integrating their experiences and learnings into their parenting style is important to give their children the best chance to experience positive wellbeing [88,89,98,108].

Feeling loved and cared for is a key aspect of wellbeing for Indigenous youth [88,98,102,103,104], with affection through physical touch referenced as a way to show such care [88,102]. Conversely, physical separation in the parent–child relationship can negatively impact youths’ wellbeing [88]. Disconnection of loved ones, sometimes through death or imprisonment, has strong and long-lasting negative impacts on youths’ wellbeing [88,104,107].

Friendships are seen as deterrents to risky behaviours and can offer a safe space for youth to discuss emotions and concerns [101,105,106,107]. Romantic relationships may result in conflict and risk to all genders, so having strong friendships outside of such connections is regarded as important to improving and maintaining healthy wellbeing [91,101,105].

Popular communication modes for youth, such as mobile phones and social media, are seen as positive for maintaining connections despite physical separation, however, they can also discourage genuine interpersonal connection [88,89,101,106]. The impacts of continuing colonial influences and racism in the lives of Indigenous communities across Australia, seen in the incursions of conflict, violence and the use of illicit substances, can negatively impact all relationship types and, subsequently, can incur on youth wellbeing through detracting from their experience of positive relationships [86,91,99,104,105,106].

##### Culture

Culture is foundational for the health and wellbeing of Indigenous youth in Australia, via the provision of strength, identity, resilience and development of meaning in life as youth grow and mature [102,108]. Cultural practices are fostered through community and kinship relationships, and knowledge sharing regarding culture and cultural activities [84,87,88,101,102]. Cultural activities and practices, such as getting out on Country [87,90,101,106], art [85,88,102], singing and music [88,102], yarning and story-telling [90,100,101], dance [88,101,103], smoking ceremonies [85,101], and fishing, hunting and bush tucker [84,87,101,106] are important aspects of Indigenous culture that improve youths’ wellbeing. Adults convey to youth the importance of passing on cultural knowledge; they teach youths about cultural identity and what their cultural practices are, instilling a sense of pride, belonging and identity for youth [88,90,98,102].

Understanding connections to Country and Dreaming (The “Dreaming” is a reference to a sacred era wherein totemic spirit beings formed the Creation and is often used to refer to an individual’s or group’s set of beliefs or spirituality [153]) is a significant component of culture for Indigenous youth [84,95,98,100,101,102,106]. Spending time on Country provides valuable opportunities to learn and connect spiritually [90], particularly with ancestral connections, who are seen to protect youth [100,104]. Where youth are not living on their own Country or colonisation has changed the natural environment, these lessons can become more difficult to share [100], thus heightening the importance of community connectedness and ceremonies to ensure cultural continuity for future generations [102].

Schools and institutions are seen as environments in which youth can engage in culture in a contemporary way [102,103]. Youths’ being accepted in an Indigenous community, regardless of their cultural knowledge, and feeling pride in their Indigeneity, strengthens their wellbeing [100,103,108]. The insidious impact of racism in Australia can, however, undermine youths’ cultural connectedness [99,102,103].

##### Aspirations for the Future

The aspirations of Indigenous youth in Australia are fostered when their foundational basic needs are met and they have strong role models who encourage pursual of opportunities in their lives [84,108] The ability to self-determine their own path in life and assume responsibilities was reported as being important to the development of their individual self-esteem and self-worth [89,98].

Indigenous communities with strong social fabrics and industries can provide critical support, employment and other opportunities for youth [82]. Accessing recreational and cultural events [87,103], vocational opportunities [105,106], and having the potential to experience life outside the community [105] all play important roles in fostering positive wellbeing of youth. The possibility of youths’ creating their own family in the future is another positive aspiration [106], however becoming a parent during adolescence may have negative impacts on youths’ ability to secure future education and career opportunities [105].

The collaboration of Indigenous and non-Indigenous community members can enable the development of further supports and opportunities for young people [82,102]. Schools that embrace Indigenous culture in the curriculum, and teach accurate Australian history, foster feelings of inclusion and acceptance, can assist in establishing identity for Indigenous youth [97,100,102,103]. Incarceration of Indigenous people [106] and substance abuse in communities [86,99,105,106], resulting from impacts of colonisation, can has deleterious effects on youths’ aspirations. Positive representation of Indigenous peoples and communities, however, particularly in mainstream media, contributes to optimistic outlooks for youths’ futures [103].

##### Identity

Feeling grounded in Indigenous culture, family and community is integral for Indigenous youth in Australia to formulate their identity and foster a sense of belonging within their communities [87,89,93,98,102,107].

Living between the two worlds of Indigeneity and Western post-settlement society can hinder youths’ navigation of connections to Country, and societal and cultural norms [86,87,100,103]. Experiences of racism, particularly regarding Indigeneity and appearance, can leave youth feeling confused and isolated, and erode identity and wellbeing [85,93,100,104]. Negative representations of Indigeneity incur on youths’ identity, inducing feelings of shame [103]. Parents identify that youth with a stronger sense of their cultural identity, and pride in their Indigenous identity [102], are more resilient to racial discrimination [108].

Strengthening youth’s connections within Indigenous communities maintains cultural and Country links, and allows for youth to explore and strengthen their individual identities as they grow [94,102]. Positive experiences of self-exploration around topics of gender, sexuality, mental health, and race all influence identity building and promote wellbeing for Indigenous youth [99,100,101,105].

##### Recreational Activities and Interests

Indigenous youth in Australia can strengthen their wellbeing whilst engaging in a range of recreational activities [82,84,86,87,88,98,99,101,103,104,105,106,108]. Sporting activities are commonly valued as an important vehicle for engaging with community [82,105], encouraging positive social behaviours [106,108], and as a way for youth to express passion and happiness whilst building strength and skills as individuals [86,88,99,101,104,105]. Indigenous athletes and sporting teams are strong conduits for feelings of pride in community and culture, especially in high profile athletes who demonstrate Indigenous excellence and achievement on the national stage [104,105]. Cultural recreational activities, like fishing, dancing and art, provide opportunities to learn about healthy lifestyles and connect with Country, identity, community and culture [87,101]. Having pets can also facilitate physical activity, and feelings of love and support, in the home environment [104]. Such activities and interests are seen to challenge boredom and provide opportunities for positive engagement [84,86,105,106,108], whilst building the foundations for a happy and healthy life [98,99,104].

##### Physical and Mental Health

Physical [83,86,87,90,95,96,97,99,103,104,106] and mental health [87,88,101,102,104,106,107] are key contributors to the experience of wellbeing for Indigenous youth in Australia. Engaging in sport [99,104] and eating healthy foods [98,104,106] facilitate physical health in Indigenous youth, whilst barriers include: overcrowded housing [83,96,98], risky sexual behaviours [86,91,102,105,106], desensitisation in communities to poor health outcomes [95,97], violence and trauma [91,106] and alcohol abuse and smoking [101,106].

Cultural wellbeing is an inextricable foundation for physical and mental wellbeing [102], with cultural activities seen to fortify health [104,106]. Kinship relations, grandparents and Elders are important figures in supporting youth to navigate issues around their physical and mental health and wellbeing [84,87,89,95,98,106]. The impact of colonisation, racism, and disrupted cultural continuity [88,89,98,102,103] on youth may manifest in poor mental health, with anxiety and depression [101,106,107], psychological distress [101] isolation [107], substance abuse [86,101], and suicide [86,101] all potential outcomes. Substance abuse, alongside little opportunity for social engagement, is seen to foster anti-social behaviours and exacerbate poor physical and mental wellbeing [99,101,106,108]. Where young people can connect to culture, have mental stimulation in areas such as schooling [104] and practice self-care [89,98], happiness and a strong spirit are built and maintained [102]. The ability to withstand racial incursions [86,98,103,104,108], remain strong and survive in the face of continuing colonial pressures, builds resilience and can ensure that youths’ wellbeing is not broken [102,103,108]. Having a trusted person to talk to, can contribute to beneficial mental health practices [106,107]. Further, the ability to access health services when needed has positive effects on the wellbeing of youth [101,106,107].

## 4. Discussion

This review provides a valuable and timely synthesis of the evidence around the aspects of life that are important to the wellbeing of Indigenous youth in CANZUS nations. Our analysis of the literature highlights the nuance between countries of aspects that contribute to experiences of wellbeing for Indigenous youth in CANZUS nations, including eight specific areas in Canada, seven in Australia, five in Aotearoa New Zealand and six in the USA. The findings of this review highlight the unique challenges faced by Indigenous youth in these nations, especially the mounting tensions found at the intersection of aspirations to maintain traditional ways of life and the experience of living in a post-colonial settlement world, that have direct implications for Indigenous youths’ wellbeing across all the CANZUS nations. Despite these tensions, the capacity of Indigenous youth to harness their cultural and personal strengths to navigate the challenges of an uncertain future offers a valuable model of wellbeing that may provide insight on how to navigate the complexity of life for all young people. For this reason, our discussion considers the similarities between CANZUS nations to highlight the commonalities and demonstrations of resilience that these diverse Indigenous youth have harnessed to survive and thrive in these two-worlds. In considering the findings across the four nations, focusing on the similarities in the parts of life that are important to the wellbeing of Indigenous youth is vitally important. These similarities can offer valuable insights into broad strategies to measure, promote and support wellbeing for Indigenous youth that can be shared and adapted globally. Similarities were apparent across CANZUS nations including culture, identity, relationships, and future thinking that all contribute to how Indigenous youth experience wellbeing and navigate living across two-worlds.

Culture and identity emerged as common themes across all nations, with strong connections to other themes of relationships and belonging. Experiences of culture and identity were strongly associated with engaging in traditional cultural activities, especially food acquisition and language. The transmission of cultural knowledge from one generation to the next was a key component of youths’ experience of wellbeing, with common incursions on this transmission including being away from traditional lands and Country, experiences of poor mental health or substance abuse, and the impact of societal pressures. Striking the balance between maintenance of traditional cultural activities and engaging in contemporary opportunities, often aligned with their future aspirations, was precarious for youth. The notion of existing across ‘two-worlds’ has been referenced for decades in relation to the experiences of Indigenous peoples’ lives post-colonial settlement [154,155]. This experience seems intensified for modern Indigenous youth: there are added pressures around climate change, technological advancement, and economic pressures [14] which must be navigated alongside the pressing need to preserve traditional cultural practices and knowledge. The presence of cultural strengths in the lives of Indigenous communities facing such issues helps to foster resilience. The finding in this review of the centrality of culture and identity to wellbeing for Indigenous youth in CANZUS nations aligns with our previous work, which highlighted that wellbeing for Indigenous adults is similarly enhanced by the strength of Indigenous identity a sense of belonging that emerged through strong cultural connection and spirituality [27,28].

The common thematic area of relationships across all nations is unsurprising, given the increasing prominence of the importance of friendships and connections with people outside of immediate family structures as children move into adolescence [156]. Our findings suggest that Indigenous youth are no exception to this general developmental stage; however, relationships may contribute to wellbeing more uniquely for Indigenous youth, as relationships within Indigenous communities are complex, incorporated within relational and collectivist understandings of wellbeing [27,28,30]. The types of relationships cited by youth were varied and included connections with parents, siblings, peers, and romantic partners, with different kinds of impacts on wellbeing occasioned from the different kinds of relationships. The challenges facing Indigenous youth across these nations associated with racism, poverty and pressures around risky behaviours were seen for many as being ameliorated by strong positive relationships with others. This has important implications for public policies and underscores the value in investing in programs and services that can support Indigenous youth to identify, engage in and maintain positive connections and relationships with others, particularly those that reinforce cultural ties. In comparison to the findings around the wellbeing of Indigenous adults in CANZUS nations [27,28], Indigenous youth referenced kinship structures less frequently, while friendships and romantic relationships more commonly arose as important relationships. This no doubt is reflective of shifting priorities and focuses across different times of life and is an important nuance to highlight when considering the changes in wellbeing for Indigenous peoples across the life course.

The shared importance of future thinking to the wellbeing of Indigenous youth across the CANZUS nations is a feature of wellbeing that is particular in nature to this age group as distinct from adults [27,28]. Our findings suggest that the weight of the future weighs particularly heavily on Indigenous youth. While uncertainties around the environment, career opportunities and the impacts of changes driven by technology are shared by many youth around the world [14], Indigenous youth must also grapple with the ongoing impacts of colonisation and racism, with impacts including diminishing access to and destruction of traditional lands and Country, and eroding community structures and values. Notably, these burdens are in some instances being offset by shifting approaches of Indigenous culture, through language and accurate representations and understanding of history being incorporated into education and employment opportunities [157,158]. These emerging signs of an increasing recognition and acknowledgment of the value of Indigenous peoples and cultures within the mainstream societies of these nations has clear and substantial impacts on the future outlook and wellbeing of Indigenous youth.

Our review identified a wide range of aspects reported in the literature as the key parts of life that contribute to wellbeing for Indigenous youth in CANZUS nations. Understanding and leveraging these parts of life within and across the Indigenous populations of these nations is critically important in supporting these young generations to face and overcome the extraordinary challenges of modern living. These findings contribute substantially to the evidence base that can enable the effective identification, measurement, and policy and program development of wellbeing for Indigenous youth. These are critical and requisite steps for ensuring that the health and wellbeing disparities stemming from colonial influences are addressed for Indigenous populations in CANZUS nations, with future Indigenous generations able to experience equitable opportunities and living conditions, and engage with activities that strengthen their wellbeing for a good life.

### Limitations and Strengths

It should be noted that this review did not include a synthesis of grey literature, which may have implications for the completeness of results. This is a particular consideration for Aotearoa New Zealand results, where searching of peer-reviewed literature in traditional Western journals and databases yielded only a small number of papers for inclusion. It is unlikely that this review has captured all contributing factors which influence the wellbeing of *tamariki* and *ranagatahi* in Aotearoa New Zealand; and there may be factors which were not captured in the remaining countries of Australia, Canada and the USA, especially Native Hawaiian youths’ residing within the USA. Further, this review yielded multiple papers from the same communities and completed by the same authors across all CANZUS nations, except for Aotearoa New Zealand. As there were specific research foci in such papers, there may be an over-representation of some factors contributing to the experience of Indigenous youths’ wellbeing in this review. Additionally, many of the included papers focused on older children, with ages below five often not the focus of the study. The elements of wellbeing which may be unique to this younger population may not have been captured.

A limitation within this review of language and framing should be noted. Indigenous populations have access to intrinsic strengths and resources that persist in the face of extremely negatively influences on their lives post-colonial settlement. Placing these strengths at the centre of discourse involving Indigenous peoples, rather than perceived deficits, is crucial in celebrating Indigenous capabilities and shifting public and institutional perceptions. Strengths-based approaches and language use are increasingly recognised as important in Indigenous research [159,160]. Our team recognises that presentation of some contributing factors to Indigenous youth wellbeing is framed negatively in the current review due to the age of the articles and efforts by our team to present data objectively, and that this may have impacts on interpretations by the reader. Our team has endeavoured to highlight the undercurrent of colonial systems impacting on Indigenous youth wellbeing where possible, and present data objectively. We hope that this review, as an initial establishment of aspects of Indigenous youth wellbeing in CANZUS countries, may provide a foundation for further research which celebrates the strengths and capacities of young Indigenous people.

While these limitations are important to acknowledge, our transdisciplinary, internationally representative, and First Nations majority investigator team is a key strength of this review. Our review included First Nations authors from Australia (AG, KN, MD, GG), Canada (AL), Aotearoa New Zealand (EW, ZA) and the USA (MC), who were able to provide guidance and direction throughout the review process. Further, the inclusiveness of this review, having no limitations on year published and searching of databases that spread across multiple disciplines and sectors, ensured inclusion of all peer-reviewed empirical literature that included aspects of wellbeing important to Indigenous youths’ of CANZUS nations.

## 5. Conclusions

Our review identified several parts of life that are important to supporting the wellbeing of young Indigenous peoples in Canada, Australia, Aotearoa New Zealand and the USA. This review makes clear that the parts of life that support and maintain strong wellbeing for Indigenous youth differs in important ways from those of non-Indigenous youth and from Indigenous adults. This makes a strong case for the development of identifiers, measures, policies and programs that target the wellbeing of Indigenous youths’ in CANZUS nations to require careful consideration of the specific age-related, cultural, social, and geographic contexts of the population of interest. The nuance between nations as evidenced in this review, underscores this point, while the commonalities in what impacts wellbeing of Indigenous youth and the demonstration of resilience that these they have harnessed in order to survive in both their cultural world and modern Western society, provide valuable insights into how information and approaches can be shared to benefit all Indigenous youth, future generations and possibly youth globally.

## Figures and Tables

**Table 1:** Medline & Pre-Medline Search Strategy.

Population	Title/Abstract search: “First Nation *” OR “First people *” OR Indigenous OR Aborig * OR “Torres Strait Islander *” OR “Torres Strait” OR “Indigenous Australia *” or “First Australia *” OR “American Indian *” OR Inuit* OR Māori* OR Maori * OR “Native American *” OR ((Canadian OR Canada) AND Aborigin *) OR “native Canadian” OR “Indigenous population*” OR Metis OR Métis OR “Alaska * Native” OR “Native Alaska *” OR “Native Hawaiian *” OR tribal
Population controlled vocabulary	MH “Indigenous peoples”
Wellbeing terms	Title/Abstract search: wellbeing OR well-being OR SEWB OR “quality of life” OR HR-QOL OR HRQOL OR QOL OR wellness OR “life quality” OR “quality adjusted life year” OR “QALY”
Wellbeing controlled vocabulary	(MM “Child Welfare”) OR (MH “Infant Welfare”) OR (MM “Quality of Life”) OR (MM “Quality-Adjusted Life Years”)
Youth terms	Title/Abstract search: child * OR children OR infant OR toddler OR ‘preschool’ OR school OR teen * OR “young adult” OR youth * OR adolescen* OR paediatric OR “young people” OR “juvenile” OR pepe OR pepi OR tamariki OR rangatahi
Youth controlled vocabulary	(MM “Adolescent”) OR (MH “Child+”)

## Supplementary Materials

The following supporting information can be downloaded at: https://www.mdpi.com/article/10.3390/ijerph192013688/s1, S1: Complete Search Strategy; S2: Screening Hierachy; S3: Review Articles Identified in Search. References [13,34,161,162,163,164,165,166,167,168,169,170,171,172,173,174,175,176,177,178,179] are cited in the supplementary materials.

## Appendix A. Nation-Specific Themes and Exemplar Quotes

**Table:**

**CANADA**
Basic Resources for Survival *“[It’s] impossible to ask a family to do something about infant development if they’re worried about where their next meal is going to come from”* [44] *[Adolescent] spoke about the difficulties of keeping up past relationships because, without money—all of which was used for food and rent—she was unable to purchase a phone card* [41] *Employment was a key issue for many people who were interviewed, particularly for younger members of the community; many were focussed on employment and business opportunities in the newly emerging diamond mining industry.* [58]
Safety and Stability *Youth reflected on the negative aspects of addictions in the health of their communities and within their schools. While some of the youth spoke to their own use of drugs and alcohol, most were in agreement that the use of substances in their own lives was incongruent with having a productive future.* [60] *[Adolescents spoke of] the anxiety and discomfort they experienced while walking down streets, standing at bus stops, hanging out in parks, and gathering outside various businesses, particularly along Main Street as police cruisers would often either slowly circle the block or pull over to the curb to interrogate them.* [62] *Given that many of the circumstances that youth found themselves in are non-consensual (e.g., child welfare, incarceration), the youth conveyed that they often had very little say in what was deemed to be in their best interests.* [55] *“All throughout my life it has been like twists and turns…. You finally get into a good home, that maybe felt like a month, and you get attached, and surprise! You’re moving!”* [45]
Relationships with Others *“I always got this vibe, I’m like “yo, there’s probably some ancestor just chilling right here, canoeing right beside me” and I thought that was awesome! I don’t know, it’s just—I always think about that. Like there’s probably someone right there just watching [out for] you.”* [54] *All of the youth agreed that listening to stories told by their grandparents and elders in the community was enjoyable and an important way of cultural knowledge transmission.* [60] *As Sandra [grandparent] told us, she wanted to be there “for their safety, so that no one can abuse them, to give them something that I had when I was growing up, a grandparent.”* [64] *“You see a lot of Aboriginal girls are pregnant nowadays…[my cousin] doesn’t want [to be pregnant] she just wants somebody she can love so that’s why she had a baby when she was really young.”* [53] *Another said that many youth follow the crowd and make choices based on what they think their peers want them to do: “That’s how it looks to me, that some kids around here, they’re tending more to want to fit into what their friends are doing than making their own choices themselves.* [46] *[Traditional counsellor] spoke of hearing young women talk about their boyfriends making suicide threats. She said that a boyfriend would sometimes threaten, “If you leave me I’m going to kill myself.”* [47]
Culture and Spirituality *“Speaking our language is important; there are more and more people using the language compared to just a few years ago. Kids are trying to use the language now too which is more meaningful for the elders”* [58] *“Spending time on the land is important for re-establishing traditions and culture for all generations, and for living by the philosophy of mino biimadisiwin (“the good life”)”* [54] *“My culture has the spirituality side of things, like the dancing and the sweats [traditional Aboriginal ceremony]. And that makes me feel more connected with myself and nature, and I like that.”* [53] *“I think we’ve found that balance between religion and spirituality. Both of them are okay. Whereas when my grandmother, my mother and I grew up it wasn’t. You were Catholics and that was that. With these kids it’s different. They’ve got both.”* [64] *“Oh yes, [the land] can help you. It can soothe you and help you take things off your mind. You can go off wooding or something like that. And take your frustrations out on a junk of wood. Or you can go out hunting and fishing … I would rather be out on the land any day rather than being in the community. It makes me feel as good as I ever feel, I think.”* [52] *[Grandmother] “We’re gonna be going home this summer, sort of like a reunion, and hopefully a lot of people will go. And I’ll take them in the canoe. They have never set foot in a river, you know, the wild river. Cook by outside. So that’s the things I am going to teach them.”* [64] *The major elements identified [for a ‘healthy community’] were food and trees/land, followed by water and culture (including language), and then school (get-togethers) and family, friendship, and community* [51] *As one youth explained, within climate change, “there’s all these other things changing—lifestyles changing, your food, your diet’s changing, the way you interact with people. What if you always go to the cabin every weekend with your family?—like that’s changing if you can’t get out. Like, climate change along with, like, so many other things in with it—interwoven in all that.”* [52] *From the time of his first sweat on the “inside,” Randle continued to seek out that positive energy and sense of spirituality and community—always wanting to help and be close to the Elders, the fire, and the cultural ways* [70]
Knowledge, opportunities and the future *In particular, traditional caregivers made the respondents aware of a traditional expectation to tend to the needs of grandchildren, and in turn to be cared for by their grandchildren in old age.* [64] *Several male interviewees also highlighted how hunting and being on the land had affected their availability or interest in pursuing other career goals and other international programs. For example, one interviewee reflected that he had been so busy and inaccessible whilst hunting that he had been unable to apply for an international exchange program with Canada World Youth and he had yet to pursue his goal of opening a maintenance shop* [40] *“I just take it day by day.” …, “I get anxiety, and I try to just go like, I don’t like thinking too much about the future. It kind of stresses me out.”* [45] *Changing economic opportunities and lifeways, shifting demographics, and technology have impacted opportunities for intergenerational engagement in St. Lewis. Older adults expressed concern about extent of changes in the community since their youth. Although they acknowledge that continuing the old way of life is no longer possible, they strongly desire to see cultural skills and values transferred to younger generations.* [57]
Identity *The connection to traditional territory “bonds the community together,” in the words of one participant. Youth in particular speak of the importance of going into the territory to “find out who you are and where you come from.”* [42] *Participants identified a range of things they would want to learn from an Elder: “I would want to know how to get an Indian name, how culture started, tradition, history, to speak my traditional language, and cooking.”* [43] *“Growing up, I always felt really out of place. I was either too brown, or too white, or too savage, or too rich. I was too skinny. I was too ugly. I was too pretty. I could never really fit in…I didn’t really grow up with a cultural background.”* [45] *“My mom has always taught me praying is what we necessarily do. I used to always see my kokum pray too. And every time I see it, I just get a feeling of pride when I see it. When I am in a room of people smudging and praying, it just gives me this sense of a cultural pride, and that’s the best feeling, gives me hope to go on, you know.”* [45] *Elders on the journey indicated that when participants have a greater sense of their history, they have a healthier relationship to their own identity.* [54] *“They were round dancing and smiling and happy. I could smell smudge, and the drums and something about being there, I was like “I belong!” I recognized that I had a community and I followed that.”* [55] *“Whenever I practice traditions I feel free, I feel like I can fly, it is an amazing feeling.”* [76]
Resilience and Independence *A crucial step for youth in reclaiming their own value lies in self-recognition. While society has long given them the message that they are irreparably damaged, youth resisted this idea and found ways to persevere and take back their lives* [55] *“There’s always going to be change … the world ain’t going to stay the same forever,” …“There are just some things where you have no control over; you can’t control weather, like you have to make the best of it and adapt.”* [52] *By using their art to challenge the lack of positive images of Indigenous people in the mainstream media, youth appeared to be taking control of their own decolonizing processes as their self-knowledge expanded.* [65]
Recreation and Interests *Yet younger Inuit, teenagers and those in their 20s, were generally very pleased with having arenas and sports available to them. Sports and related activities were associated with happiness and health for many in this age group.* [48] *While it was at times difficult for youth to explain why they produced the art that they did, it is evident they were drawn to more symbolic means of self-expression that asserted their identities as Indigenous people and maintained cultural continuity.* [65]
AUSTRALIA
Basic Needs *“my son does not feel safe here and is constantly on the lookout for signs of trouble—I feel it’s unfair that he is so on edge because of where we live”.* [98] *“Sometimes after school, me and my Grandad go swimming. We take the big air tanks and we take spear guns and we see holes, we think they’re crab holes in there” (9-year-old boy FG2).* [87] *Participants said some people experiencing homelessness were eligible to stay in temporary state-provided accommodation, including low-cost motels, caravan parks or boarding houses. However this was described as incredibly stressful, often involving frequent moving between poorly located placements (no transport, services) that were often described as unsuitable for children,* *“She can’t take baby to the doctors, she can’t go to the shops to get milk if she needs it. Some of the places that they’re putting the Mums haven’t got cooking facilities… she’s got a young baby, and she can’t even warm up a bottle of milk, and that’s where a lot of them are”* [83] *Carers felt that a stable and structured home environment including children having a “roof over their heads”, and that having a regular routine, getting sufficient sleep and maintaining good hygiene was important for children’s health and wellbeing.* [98]
Relationships *“He [my Uncle] takes me fishing, he teach me how to, like, spearfish with spear, and when we catch them, peel the skin off”. This statement was followed by the expression of how the activity made the participant feel: “Really, really, really, good”* [87] *For the children of [town], the benefits of connectedness appeared extensive, resulting in there being a natural scaffolding to support, protect and guide them within the community, enabling them to encounter high levels of social cohesion and social capital, enriched by the relationships and social contacts that they and their immediate families experienced in the town.* [82] *“These kids need to get back on their Country and spend time with their Elders and you will find more better-focused kids better-behaved kids.”* [102] *“It’s a strict family, the girls are not allowed to make trouble with boys.”* [105] *“Another impact is that we over-parent. We want to make everything alright, want to try and fix everything for them. It’s like you become controlling … without realising it. [We] need to let them make their own mistakes and learn from them.”* [89] *Intimate relationships for young women were characterised by their lack of autonomy* *and their perception that their boyfriends will move on to another partner if given sufficient opportunity. Girls risked well-being, security and the relationship if they refused sex, whereas young men were expected to move from one relationship to the next* [91] *“[He has a] broken heart… he just got dumped—girlfriend problems” “… girls get jealous and start fights, yeah that is like most of the fights I have had…”* [101]
Culture *Promoting this connection to Country was identified as critical to Aboriginal child wellbeing, despite challenges of fostering this connection when many urban Aboriginal people did not live on their own Country. Participants described strategies of teaching children the geographical boundaries of their Country, visiting Country, telling stories about experience of previous generations, and teaching children about significant places or plants for medicine and tools.* [102] *“Being a Koori kid is you can be proud of yourself sometimes because you do things for your culture. I was proud of myself one time cause we were talking about cultures at my old school and that started with my Aboriginal culture.”* [104] *“access to culture and cultural activities—it all grows from there—family, health, confidence, to be part of a bigger picture”* [98] *“I’ve had times when I’ve been on Country by myself and times when I’ve been with family as well and I think it’s really special to feel like you’re being looked after, you know, that you’re really safe, I mean it’s the one place I can go and really feel safe”*—[100]
Aspirations for the Future *“Probably breaking into places and sniffing [petrol]. That was horrible. And, people doing drugs was common when we were growing up. They survived it—some people didn’t. It was hard growing up in a community. Everyone knows you because it’s so small. Tight knit families. Death would affect everyone, which was no good.”* [106] *Children articulated their aspiration to further learn about traditional Indigenous lifestyles, including: “What Aboriginal people ate” (8-year-old girl FG7), “How they survived in the deserts” (11-year-old boy FG4), “I want to learn how they lived… so I know more about them” (10-year-old girl FG6).* [87] *“encourage our kids to have a go at different things, to enhance their natural skills and ability and to shine through their own personal self-esteem, self-concept and self-worth”.* [98] *“I think it’s still really weird to hear that in secondary school’s that Indigenous culture is still not being taught, like that really frustrates me at this day and age that kids only get 1788 the first fleet came and that’s where Australia started. That just does my head in.”* [100]
Identity *“Kids don’t have a sense of belonging or who they are…so when there are elders or other people in the community who can let them know where they’ve come from, that really helps them…(Father)”* [107] *“This link between a sense of identity and selfesteem was considered particularly important for Aboriginal children with light-coloured skin who may not be immediately identified by others as being Aboriginal. Knowing that inside it doesn’t matter the colour of their skin they are all Kooris … we’ve always just told the kids you know it doesn’t matter how light or how dark your skin is, it’s what you feel in here.” (Sharni)* [102] *“[Cultural education] gives children an insight on how special they are, and the answers to origins … the balance they need to grow in this life”.* [98] *Pride in Aboriginal identity was often portrayed as the opposite of being ashamed, and an important protective factor for Aboriginal children from the negative effects of racism.* [102] *“I’ve sort of grown up in this really, really, white school…and you come into a place where everything is like black, black, black, black, it’s a slap in the face, and put it this way, I am scared to say stuff around my cousins because I don’t [know] what’s right and what’s wrong.”* [100]
Recreational Activities and Interests *Recreational groups and organisations were identified as playing key roles, serving as vehicles for enhancing communication and the wellbeing of children, in particular sporting clubs and events, which acted as opportunities for town-based and farming families, Aboriginal and non-Aboriginal communities, to join together.* [82] *Fishing connected children to healthy lifestyle behaviours such as physical activity and healthy eating. Children frequently acknowledged that they as a family would consume what they caught and enjoyed participating in these activities. F: “Did you cook it up?” P: “Yes me and my Dad did. My Dad guts it and that and put the scales off and I cut it up” (9-year-old boy FG2).* [87] *“I think keeping children actively involved in sports is a major part in their health and wellbeing”. The longterm benefits of an active lifestyle so that children “can achieve their dreams and [be] healthy, fitter adults” was recognized* [98] *“Sport… releases endorphins which make you happy” “Get things off your mind… go for a walk, swimming, exercise, training”* [101] *‘Playing footy makes me feel healthy and well’ (Interview)* [104]
Physical and Mental Health *“He helped me eat my vegetables…stop being lazy…get up and do something…get some exercise, go for a walk or go fishing’”* [106] *‘Grandparents, they tell you their mistakes and their health issues and they tell you what not to do…they tell you from their life, growing up’* [106] *“community are actively involved/aware of the cause of issues that underpin many of the problems in our community, which impacts our children’s development. When issues get addressed, we will make a significant improvement to our children’s lives and futures”* [98] *“I think it’s very important even at a young age, to be happy, to see a young child smile”. (Sally)* [102]
AOTEAROA NEW ZEALAND
Belonging, Care and Support *“Because it’s [wahakura] a flax Māori feel, you know, so they feel right from the start that it’s part of them that it’s a part of their whānau. So they’re really proud when they say that they have a wahakura.”* [110] *Ngaire employed an open door policy, so she usually prepared ample amounts of kai (food) to ensure anyone who arrived at meal time would be fed: ‘She [Ngaire] cooked a very large meal so there would be leftovers…but the moko’s [grandchildren] all came for dinner (7 people in total) and most of it got eaten…’ [Household 3, visit 6 field note]* [112] *“If you learn about the environment, how the moon works, and all of that, then you are becoming more self-determining as an individual, also as a family, and as a tribe and subtribe … So that’s the bigger picture for us”.* [115] *Mother: Yeah and then we went down and took my younger sister with us but they told her that she wasn’t allowed to see him because she’s eight years old so my little sister came all the way down and wasn’t even allowed to see him. Grandmother: And that was one of our concerns, they said it can only be a direct sibling. Mother: Yeah, only direct siblings are allowed to see the babies. Grandmother: To me that’s the White way of thinking, we’re Māori.* [111] *Probably just like being around each other and like making lots of memories so our kids can tell them what we do, and so it like just keeps going.* [116]
Ahurea (Culture) *Older key informants considered harakeke and therefore the wahakura to have tapu (sacred) and rongoā (healing) qualities which enhanced infant wellbeing at all levels. The harakeke was perceived to emanate “warmth” that the baby was nurtured by. They referred to the wahakura as a “living thing”, meaning that it had an innate vitality and spiritual value.* [110] *Gardening enabled whanau to remain connected to the whenua (land), providing satisfaction of looking after the whenua so it could provide nutritious kai for themselves and others. Households with surplus produce could share this healthy food with others and express manaakitanga. Thus, gardening contributed positively to the expression of hauora (well-being).* [112] *As the centre of whanau, infants embody the hopes and aspirations of their ancestors both past and present, and represent taonga tuku iho—gifts that are passed down through generations. Infants are crucial to the continuance of whakapapa, and, therefore, the vitality of whānau, hap¯u, and Iwi. As such, practices that hold space for infants—physical intimacy, nurturing, spiritual safety, and connecting with place—were ways that these whānau enacted agency as wh¯anau.* [111]
mātauranga (knowledge) and mōhiotanga (knowing) *Passing on food knowledge and skills to future generations was an important goal for most participating households.* [112] *“[In] te ao Māori there is history of wānanga/ marae-based learning … Learning that their tupuna (ancestors) and themselves are all ‘cultural scientists’ in their own right and they have the potential to excel and attend university to pursue a career in fields of science, Pūtaiao, environmental/ taiao.”* [115]
Identity and Agency *For example, some felt that [wakahura] encouraged the baby to become independent, a few even suggesting that bedsharing made the baby too dependent on the mother.* [109] *For some choosing a Māori name was a deliberate move away from the recent historical trend of using Pakeha names and a means of ensuring their baby had a strong Māori identity.* [109] *Participant 6 spoke of an aim to: reaffirm their sense of belonging, cultural identity, knowing who they are so they are able to stand proud but humble in this ever-changing world. That they may do so with a consciousness of knowing the delicate interconnectedness of themselves and others with their local and global environment.* *Mother: They’ve been saying that our baby is the baby with no name because we haven’t named him yet. We have named him but we don’t want to share that straight away. It’s important to us, it’s special to us.*
Physical Health *Starts from when the kids are small…giving them the right food choices versus KFC, McDonalds, so then the kids know when they’re older, they have the option of a healthy food choice…’ [Household 2, visit 14, mother’s quote].* [112]
USA
Safety and Basic Needs *“If we could get people jobs away from [the community], but two weeks on, two weeks off, so they can come home for a little while. Because a lot of people don’t like to move away from here. But there are so little jobs here”.* [122] *In focus groups, they easily rattled off the additional stressors, such as “finding a place to stay,” “…bills, cleaning the house and having a job,” and “voting even though we don’t know how.”* [140] *A younger boy explains, “Whenever [friends’] dad’s not doing good [drinking], [friend] comes to our house and we take care of him.”* [143] *“And my big concern is, the kids in this neighbourhood you know, we saw three shootings out here about 6 months ago. One young man died on that doorstep over there. That makes an impact on you, you know. So it is like gangs and drugs are eating our children up and spitting them out and putting them into incarceration. They do not belong there. What I see is, it takes a whole village to raise a child. And I think we try to do that.”* [138]
Relationships and Connection *“[I] hang out with the people I know are going to stay away from it…that [means] I know I’m gonna stay away from drugs and alcohol. I try to stay with them as much as possible so I don’t have to go home.”* [140] *“For one, we value our blood, we’re so few, we do everything we can to make sure we keep the bloodline going. For eternity, as far as I’m concerned. So that child that’s born, whether it’s here or on, somewhere else, there should be something saying, ‘‘Look you have another member of the community.’’”* [118] *“When I’m not doing anything, or like if I have chores, sometimes she’ll (friend) come over and help me, and if she has chores, I’ll go over and help her. Or if I need help with my homework, she’ll help me or if she needs help, I’ll help her. We’re just kind of like there for each other.”* [142] *Many youth also highlighted how their experience and relationships on reservations provided important insights in Native culture, contemporary Native status, and their community needs. For some, this helped spark a strong pride and connection to their Native identity and for others served as motivation for their own success, driven by an ultimate desire to “give back” or make things better.* [144]
Culture and Tradition *“I don’t know most of my tradition and…I get really upset sometimes that I can’t even speak my own language. I can’t speak it fluently.”* [127] *“The key for the modern Yup’ik is to ensure that our children succeed in the Western school system while at the same time teaching them traditional cultural values that have stood the test of time.* [117] *“I’ve had kids where they’re like, you’re not gonna be able to get them for more than five minutes…And they sat there, I’ve done dreamcatchers, medicine bags. They—they do these things, they actually pay attention and they do what they have to do and they’re so proud of it, and they hold onto these things, you know? Just from these activities”* [118] *“[My aunt] taught me how to cook like all the Native dishes … it’s like all these vegetables like squash and corn, just really good. It’s good. Then you put whatever seafood you want. She taught me how to weave baskets. She just like tells me a lot of stories that like has been passed down I guess.”* [132] *“For off-reservation youth, relationships with people on the reservation were also associated with a sense of comfort and acceptance, and were vital for helping them maintain connections to Native culture, despite spending most of their day-to-day lives in non-Native environments and with non-Native people.”* [144] *“We have our ties here. Twenty years down the road this will always be our home. This is where our ancestors were.”* [130] *“Being a good Indian and to exist in the White world with those values, those materials values, now that’s a conflict.”* [135] *‘‘I don’t want him to be stuck in this culture, I want him to be part [emphasis added] of it... It’s not that you’re born Native so you have to do this kind of stuff.’’* [137] *“To me [culture] means having hunting skills that shows up a lot and like picking berries and like harvest skills and teaching kids how to do stuff. It doesn’t matter like what you’re teaching, but as long as it’s positive and you’re making a good impression on the next generation then I think that’s an Inupiat thing that we all do.”* [141] *“Just got to see the reality for what it is. You stay here [in the traditional community] and you feel, like, trapped and you’re alone. And then when you get to town it’s the same thing. There’s a lot of people there, but you still feel alone because most people—they act totally different from how we act. We respect our elders. Them? They don’t really respect anybody.”* [144]
Cultural Identity and Pride *Another participant spoke to the searching for belonging and identity that a child feels: It hurts the child because they’re continuously looking for something, you know something to belong, they’re looking for their identity, they’ve got a big hole there.”* [118] *Identity among Indigenous peoples is far more in-depth than cultural values and uniqueness as a people. Identity for Onkwehonwe youth is understanding the connection to and significance of place. It involves understanding the importance of relationship and the responsibility we have on a personal and collective level to the Earth and universe, and how this, in turn, affects us as a people.* [125] *While today’s youth rarely speak more than a few words in their traditional Inupiaq language, this school represents such a movement towards ‘‘recovering Native identity’’ (Paul’s words, again) through the relearning of these ‘‘cultural treasures.’’* [137] *“We have language class here every Thursday… my brother, sister, and I really like it. My uncle can speak it. A lot don’t know the language, but some are still fluent. I think it really helps because we still have the museum and we have a lot ofelders that teach that stuff, we still have the birdsinging, and jingle-dancing. I think it’s very cool that we’re still into that.” (Wood 2018)* *“I have grown more spiritually over the years by reconnecting to the people, to the land, to the spirit and myself. I started to be more real. You feel yourself as Onkwehonwe, just in that description is … what it means … your “real self””.* [125] *They [Inupiat] seem stronger back then and more traditional. Their language was there. It’s way different. I don’t think it’s the same. Maybe same, like, how it was in their community, and with their family. But I don’t think it’s the same.* [137] *“[Being Inupiaq] means a lot because I get to hunt and do lots of stuff that most people don’t get to do.”* [142]
Looking to the past and future *“Parents and elders voiced concerns and remembered their own difficult times with school”* [135] *“I mean, to even go to high school off the rez, that takes like a lot because for some people the rez is all they’ve known, so it takes all they have to leave.”* [130] *“As young people, it has been hard for us to understand our role today. Most of us do not speak or even understand our Inupiaq language, so we don’t communicate well with our parents and grandparents. On top of that, we have so much idle time due to the lack of jobs in our villages, most of us don’t have to worry about getting water, wood, taking care of dogs, and getting caribou and fish and other subsistence foods takes a lot less time now. We go through twelve years of school, but most of us aren’t prepared to succeed in college or other training, and therefore the many jobs in the region are not filled by people from here.”* [139] *“It’s a way of life that you can pass down to your children, in a way that you can’t in the city or in a small town…not only are you passing down how to hunt and fish but you’re also teaching different family morals as well.“* [123]
Being Healthy *Several key themes emerged from the analysis of transcripts, and they revealed participants’ appreciation of traditional ways and subsistence activities as integral to the unique quality of life in rural communities, as well as central to efforts to prevent suicide. Traditional ways were thought to facilitate important relationships, promote healthy living, contrast with contemporary challenges and communicate important cultural values.* [123] *Yeah, [historical trauma is] affecting them. When you go out there and you see these little kids, and then when you compare the way these little kids act to the ones that you see in town, they act different.…Some of the little kids in [tribal community], they kind of act bad because they’re like two, and they’re already cussing, and stuff like that. When you see a little kid that’s two years old in town, they just, like they’re happy, and then the other one’s all mad and stuff like that.* [127] *‘‘I guess healthy eating, we have fish, we have a lot of fish. Like I said, there is some subsistence fishing, people go hunting so there’s fresh meat. I guess that’s one aspect of healthy living’’* [123] *We go [to the Reservation] once or twice a year. It’s such a relief. It’s comfy. It’s beautiful. You still have all the same worries, financially and family wise, but it’s more comfy and there’s more of a sense of home.* [144]
