# Supplementary material for: Aspects of Wellbeing for Indigenous Youth in CANZUS Countries: A Systematic Review
Source: Int J Environ Res Public Health. 2022 Oct 21;19(20):13688. doi: 10.3390/ijerph192013688 (PMC9602510; doi:10.3390/ijerph192013688)
Supplement: Supplementary file 1 [file ijerph-19-13688-s001.zip › Supplement1_220607.pdf]

<u>Supplement Table S1</u>

<table>
<tr><th>Database</th><th>Indigenous population</th><th>Subject Headings</th><th>Wellbeing Terms</th><th>Subject Headings</th><th>Youth Terms</th><th>Subject Headings</th><th>Limiters</th></tr>
<tr><td>APA PsycINFO via EBSCOhost</td><td rowspan="5">“First Nation*” OR “First people*” OR Indigenous OR Aborig* OR "Torres Strait Islander*" OR "Torres Strait" OR "Indigenous Australia*" OR "First Australia*" OR “American Indian*” OR Inuit* OR Māori* OR Maori* OR “Native American*” OR native OR “native Canadian” OR eskimo OR “Indigenous population*” OR Metis OR Métis OR “Alaska* Native” OR “Native Alaska*” OR “Native Hawaiian*” OR tribal OR ((Canadian OR Canada) AND (Aborigin* OR "First Nation*" OR Inuit*))</td><td>DE "Indigenous populations"</td><td rowspan="5">wellbeing OR well-being OR SEWB OR “quality of life” OR HR-QOL OR HRQOL OR QOL OR wellness OR “life quality” OR “quality adjusted life year” OR “QALY” (TI/AB)</td><td>"Quality of Life" OR "Health Related Quality of Life" OR "Well Being"</td><td rowspan="5">TI/AB child* OR children OR infant OR toddler OR 'preschool' OR school OR teen* OR "young adult" OR youth* OR adolescen* OR paediatric OR "young people" OR "juvenile" OR pepe OR pepi OR tamariki OR rangatahi</td><td>-</td><td rowspan="5">Human; peer - reviewed (where available in database searching interface).</td></tr>
<tr><td>CINAHL PLUS via EBSCOhost</td><td>MH "Indigenous peoples"</td><td>(MM "Quality of Life+") OR (MM "Quality-Adjusted Life Years") OR (MM "Psychological Well-Being")</td><td>(MM "Adolescence") OR (MM "Child+")</td></tr>
<tr><td>Embase</td><td>indigenous people'/exp</td><td>'psychological well-being'/exp OR 'quality of life'/exp</td><td>'child'/exp OR 'adolescent'/exp</td></tr>
<tr><td>Medline & Pre-Medline via PubMed</td><td>MH "Indigenous peoples"</td><td>(MM "Child Welfare") OR (MH "Infant Welfare") OR (MM "Quality of Life") OR (MM "Quality-Adjusted Life Years")</td><td>(MM "Adolescent") OR (MH "Child+")</td></tr>
<tr><td>PUBMED</td><td>Indigenous Peoples[Mesh]</td><td>"Quality of Life"[Mesh]) AND "Quality-Adjusted Life Years"[Mesh]</td><td></td></tr>
</table>
