# Supplementary material for: Aspects of Wellbeing for Indigenous Youth in CANZUS Countries: A Systematic Review
Source: Int J Environ Res Public Health. 2022 Oct 21;19(20):13688. doi: 10.3390/ijerph192013688 (PMC9602510; doi:10.3390/ijerph192013688)
Supplement: Supplementary file 1 [file ijerph-19-13688-s001.zip › Supplement2_220607.pdf]

Supplement Document S1

INDIGENOUS YOUTH WELLBEING SYSTEMATIC REVIEW – SCREENING HIERARCHY

Please ensure you look at each TI/AB in the order shown in the hierarchy table below. This will increase the consistency of exclusion reasons as a group ready for reporting.
For example, if you use the exclusion reason "wrong study design" this means you have already checked first that it is in English, second it is the correct publication type, and third it is the correct population BEFORE tagging as "wrong study design".

Inclusion criteria: peer reviewed, reporting new empirical data, including qualitative results, reports on the factors of wellbeing and quality of life for the selected Indigenous youth populations within Australia, Canada, New Zealand and the United States, young person themselves reports, proxy for the young person reports.

Exclusion criteria: grey literature, poster abstracts, newspaper articles, medical case reports and dissertations, published languages other than English, and if the paper does not report separately on findings for Indigenous people.

Exclusion reason	If . . .
**1. foreign language**	Not English (most should be English)
**2. wrong publication type**	Grey literature, poster abstracts, newspaper articles, medical case reports and dissertations, opinion piece, position statement
**3. wrong population**	Not Indigenous youth (<18 years) in Australia, Canada, New Zealand and the United States – this population needs to be a stated focus; okay if sub population
**4. wrong study design (non-qualitative)**	No mention of qualitative results or new empirical data in abstract, and non-relevant reviews
**5. disease/condition specific focus**	If the study cohort has a focus on one specific disease/condition, or the results are specific to the context of that disease/condition and not applicable to the general population, exclude.<br><If the cohort focused on more than one disease, and passes the exclusions below, include and add the word "disease" in the label box>
**6. Systems/setting focus**	If the study is looking too narrowly at a specific system, setting or topic– for example a health service or program evaluation – then too narrow for our review
**7. nil wellbeing focus**	Doesn't report factors of wellbeing or quality of life
**8. review**	Systematic or other reviews relevant – for checking ref lists

Other labels	If…
- **Proxy**	The study uses a proxy (eg. Parent, guardian, caregiver, teacher, etc) to report on wellbeing of the young person
- **Under 5**	The study reports on the wellbeing of children under the age of 5
- **Background**	The study is relevant and could be used as background for the manuscript but is not included in the review overall based on the above exclusion reasons

Three buttons	
Maybe	Choose this button for ones you want to discuss with the group – especially good for ones that aren't clearly in or out
Include	Any that pass all the reasons above (clearly in) or that you need to see the FT to check anything, include for FT review
Exclude	Choose this button when your article doesn't pass one of the exclusion reasons above and use the "reason" in the box immediately to the right of this button to tag the reason from the hierarchy above
