# Supplementary material for: Aspects of Wellbeing for Indigenous Youth in CANZUS Countries: A Systematic Review
Source: Int J Environ Res Public Health. 2022 Oct 21;19(20):13688. doi: 10.3390/ijerph192013688 (PMC9602510; doi:10.3390/ijerph192013688)
Supplement: Supplementary file 1 [file ijerph-19-13688-s001.zip › Supplement3_220524.pdf]

Document - Supplement S3

Reviews identified in the initial search, which were mined for eligible referenced articles.

1. Adelson N. The embodiment of inequity: health disparities in aboriginal Canada. Canadian journal of public health = Revue canadienne de sante publique. 2005;96 Suppl 2:S45-S61.
2. Akbar L, Zuk AM, Tsuji LJS. Health and Wellness Impacts of Traditional Physical Activity Experiences on Indigenous Youth: A Systematic Review. Int J Environ Res Public Health. 2020;17(21).
3. Azzopardi P, Kennedy E, Patton G, Power R, Roseby R, Sawyer S, et al. The quality of health research for young indigenous australians: Informing health priority, intervention and future research need. Turk Pediatri Arsivi. 2013;48:74.
4. Azzopardi PS, Sawyer SM, Carlin JB, Degenhardt L, Brown N, Brown AD, et al. Health and wellbeing of Indigenous adolescents in Australia: a systematic synthesis of population data. Lancet. 2018;391(10122):766-82.
5. Barros N, Tulve NS, Heggem DT, Bailey K. Review of built and natural environment stressors impacting American-Indian/Alaska-Native children. Reviews on environmental health. 2018;33(4):349-81.
6. Bombak AE, Bruce SG. Self-rated health and ethnicity: focus on indigenous populations. International journal of circumpolar health. 2012;71:18538.
7. Bombay A, Matheson K, Anisman H. The intergenerational effects of Indian residential schools: Implications for the concept of historical trauma. Transcultural Psychiatry. 2014;51(3):320-38.
8. Hawkins EH, Cummins LH, Marlatt GA. Preventing Substance Abuse in American Indian and Alaska Native Youth: Promising Strategies for Healthier Communities. Psychological Bulletin. 2004;130(2):304-23.
9. Hishinuma ES, Smith MD, McCarthy K, Lee M, Goebert DA, Sugimoto-Matsuda JJ, et al. Longitudinal Prediction of Suicide Attempts for a Diverse Adolescent Sample of Native Hawaiians, Pacific Peoples, and Asian Americans. Arch Suicide Res. 2018;22(1):67-90.
10. Jongen C, McCalman J, Bainbridge R, Tsey K. Aboriginal and Torres Strait Islander maternal and child health and wellbeing: A systematic search of programs and services in Australian primary health care settings. BMC Pregnancy and Childbirth. 2014:251.
11. Kairuz CA, Casanelia LM, Bennett-Brook K, Coombes J, Yadav UN. Impact of racism and discrimination on physical and mental health among Aboriginal and Torres Strait islander peoples living in Australia: a systematic scoping review. BMC Public Health. 2021;21(1):1302.
12. Kilian A, Williamson A. What is known about pathways to mental health care for Australian Aboriginal young people?: A narrative review. International Journal for Equity in Health. 2018;17(1).
13. Kirmayer LJ, Brass GM, Tait CL. The mental health of Aboriginal peoples: transformations of identity and community. Can J Psychiatry. 2000;45(7):607-16.
14. Kirmayer L, Simpson C, Cargo M. Healing traditions: Culture, community and mental health promotion with Canadian Aboriginal peoples. Australasian Psychiatry. 2003;11(Suppl1):S15-S23.
15. Liu DMKI, Alameda CK. Social determinants of health for Native Hawaiian children and adolescents. Hawaii medical journal. 2011;70(11 Suppl 2):9-14.

16. Lucero NM, Leake R. Expressions of Culture in American Indian/Alaska Native Tribal Child Welfare Work: A Qualitative Meta-Synthesis. Journal of Public Child Welfare. 2016;10(3):327-47.
17. MacPhail C, McKay K. Social determinants in the sexual health of adolescent Aboriginal Australians: A systematic review. Health & Social Care in the Community. 2018;26(2):131-46.
18. Priest N, Mackean T, Waters E, Davis E, Riggs E. Indigenous child health research: a critical analysis of Australian studies. Australian & New Zealand Journal of Public Health. 2009;33(1):55-63.
19. Rountree J, Smith A. Strength-based well-being indicators for Indigenous children and families: A literature review of Indigenous communities' identified well-being indicators. American Indian and Alaska Native Mental Health Research. 2016;23(3):206-20.
20. Sheppard AJ, Hetherington R. A decade of research in Inuit children, youth, and maternal health in Canada: areas of concentrations and scarcities. International journal of circumpolar health. 2012;71:18383.
21. Smallwood R, Woods C, Power T, Usher K. Understanding the Impact of Historical Trauma Due to Colonization on the Health and Well-Being of Indigenous Young Peoples: A Systematic Scoping Review. J Transcult Nurs. 2021;32(1):59-68.
